# Efficient up-conversion in Yb:Er:NaT(XO_4_)_2_ thermal nanoprobes. Imaging of their distribution in a perfused mouse

**DOI:** 10.1371/journal.pone.0177596

**Published:** 2017-05-18

**Authors:** Carlos Zaldo, María Dolores Serrano, Xiumei Han, Concepción Cascales, Marta Cantero, Lluís Montoliu, Elvira Arza, Valeria R. Caiolfa, Moreno Zamai

**Affiliations:** 1 Instituto de Ciencia de Materiales de Madrid, Consejo Superior de Investigaciones Científicas (ICMM-CSIC), Madrid, Spain; 2 Departamento de Biología Molecular y Celular, Centro Nacional de Biotecnología, Consejo Superior de Investigaciones Científicas (CNB-CSIC), Madrid, Spain; 3 CIBERER, Instituto de Salud Carlos III (ISCIII), Madrid, Spain; 4 Unidad de Microscopía e Imagen Dinámica, Centro Nacional de Investigaciones Cardiovasculares (CNIC), Madrid, Spain; 5 Centro di Imaging Sperimentale, Ospedale San Raffaele, Milano, Italy; Institute of Materials Science, GERMANY

## Abstract

Yb and Er codoped NaT(XO_4_)_2_ (T = Y, La, Gd, Lu and X = Mo, W) disordered oxides show a green (Er^3+^ related) up-conversion (UC) efficiency comparable to that of Yb:Er:β-NaYF_4_ compound and unless 3 times larger UC ratiometric thermal sensitivity. The similar UC efficiency of Yb:Er doped NaT(XO_4_)_2_ and β-NaYF_4_ compounds allowed testing equal subcutaneous depths of *ex-vivo* chicken tissue in both cases. This extraordinary behavior for NaT(XO_4_)_2_ oxides with large cutoff phonon energy (ħω≈ 920 cm^-1^) is ascribed to ^4^F_9/2_ electron population recycling to higher energy ^4^G_11/2_ level by a phonon assisted transition. Crystalline nanoparticles of Yb:Er:NaLu(MoO_4_)_2_ have been synthesized by sol-gel with sizes most commonly in the 50–80 nm range, showing a relatively small reduction of the UC efficiency with regards to bulk materials. Fluorescence lifetime and multiphoton imaging microscopies show that these nanoparticles can be efficiently distributed to all body organs of a perfused mouse.

## 1. Introduction

The development of nanoscience and nanotechnology has opened new challenges for the measurement of physical properties at the nanometer scale and for actuation on nano- or micro-metric systems. An often used method for these purposes is the incorporation of inert nanoparticles (NPs) in thermodynamic equilibrium with the unknown system, acting as probes. Within this approach, NPs with up-conversion (UC) properties are recognized fluorescence nanoprobes because they are free of photobleaching shown by organic dyes [1] or photoblinking of semiconductor quantum dots,[2] and their excitation in the near infrared (NIR) circumvent the problem of undesired tissue and cell autofluorescence typically observed under ultraviolet/blue photoexcitation.[3] Therefore, UCNPs have been extensively proposed for thermal nanosensing,[4,5] optical nanoencoding,[6] photocatalysis,[7] subcutaneous photodynamic therapy,[8,9] biosensing,[10] cancer cell recognition and treatment,[11] intracellular drug delivery,[9,12] as well as for *in vitro* and *in vivo* biomedical imaging. [9,13,14]

A further outstanding characteristic of UCNPs is their intrinsic multifunctionality: An optical image can be obtained simultaneously to the measurement of a physical property (most typically temperature when using Er^3+^ green UC). When combined with magnetic properties provided by the incorporation in the host of a paramagnetic ion, like Gd^3+^, optical and magnetic resonance multimodal imaging is possible.[15,16] Further, UCNP multifunctionality can also be obtained by surface functionalization with specific molecules for a given target. UCNP composites based on fluorides have been, in fact, extensively studied for cancer cell recognition and treatment, and surface functionalizing methods have been described for them. [10,11,17]

Although UC is a phenomenon observed for different Ln (Ho, Tm, Er, etc) in virtually any Ln doped solid, Yb:Er:β-NaYF_4_ has become the material of reference for UCNP fabrication because up to now it is the reported compound with largest UC efficiency.[18] In this system, Yb^3+^ absorbs the excitation light near to ≈980 nm (^2^F_7/2_→^2^F_5/2_ transition) and transfers the absorbed energy to Er^3+^ by energy diffusion through the crystalline host. The origin of the singular UC efficiency of Yb:Er:β-NaYF_4_ has been attributed to the combination of good matching of Yb and Er energy level gaps, low host cutoff phonon energy (ħω≈ 500 cm^-1^), and presence of multisites for the Ln,[19] although the relative importance of these contributions is not yet fully clear. Thus, based in this merit, the large number of reviews on the state of art about the design, and applications of UCNPs have β-NaYF_4_ as the almost unique protagonist host.[20–23]

Despite the prevalence of Yb:Er:β-NaYF_4_ as UC media, the search of alternative UC materials is continuously pursuit due to the following reasons: i) The synthesis of β-NaYF_4_ hexagonal gagarinite phase ($P\bar{6}$ space group) competes with the nucleation of the substantially less efficient α-NaYF_4_ cubic phase ($Fm\bar{3}m$ space group); ii) preparative routes for fluorides are often environmental harmful; iii) the UC ratiometric thermal sensitivity (*S*) of Yb:Er:β-NaYF_4_ is weak, in comparison to other studied materials; [24] and iv) the UC efficiency of Yb:Er:β-NaYF_4_ is reduced by three orders of magnitude on going from bulk to ultrasmall NPs with ≈10 nm size.[25]

Oxides have been studied as an alternative for UC purposes because of their superior chemical stability, easy synthesis and in most cases absence of phase transformations or polymorphism. However, after numerous studies it is generally believed that oxides with large cutoff phonon energy (typically ħω≈ 800–1000 cm^-1^) can not achieve green Er^3+^ UC efficiencies comparable to that of fluorides because the ^2^H_11/2_+^4^S_3/2_ Er^3+^ electron population in oxides is strongly depleted by non radiative multiphonon relaxation to the low lying ^4^F_9/2_ Er^3+^ mutiplet, giving rise to strong red UC. This situation is also encountered for Yb:Er:Lu_2_O_3_ despite its lower cutoff phonon energy (ħω ≈ 650 cm^-1^).[26]

The present work is motivated by recent reports of large UC ratiometric thermal sensitivity (*S*) in four members of the MT(XO_4_)_2_ (M = Li^+^, Na^+^ or Ag^+^, T = Y^3+^, Bi^3+^ or any trivalent lanthanide Ln^3+^, and X = Mo^6+^ or W^6+^) tetragonal double molybdate (DMo) and double tungstate (DW) compound family: 10at%Yb:1at%Er:NaY(WO_4_)_2_, *S*(317 K) = 112×10^−4^ K^-1^;[27] 40at%Yb:2at%Er:AgLa(MoO_4_)_2_, *S*(317 K) = 120×10^−4^ K^-1^;[28] 5at%Yb:2at%Er:NaY(MoO_4_)_2_, *S*(317 K) = 77×10^−4^ K^-1^;[29] and 10at%Yb:1at%Er:NaGd(MoO_4_)_2_, *S*(317 K) = 160×10^−4^ K^-1^,[30] in comparison with the value reported for 20at%Yb:2at%Er:β-NaYF_4_, *S*(317 K) = 35×10^−4^ K^-1^.[31]

Large UC ratiometric thermal sensitivity of used nanoprobes is an essential, but not sufficient, condition for any envisaged UCNP application, being large UC efficiency and suitable NP size simultaneous requisites. As previously indicated, the presence of Ln^3+^ multisites and/or structural disorder leading to a spatially variable distribution of crystal field potentials around Ln^3+^ are though to be key factors contributing to the large UC efficiency, and these structural characteristics are certainly shared by hexagonal β-NaYF_4_ and tetragonal ($\bar{I4}$ space group) scheelite-like MT(XO_4_)_2_ hosts, i.e. in the latter two different crystal sites, 2*b* and 2*d*, are randomly occupied by Na, T and Yb/Er cations. Neither earlier indicated works dealing with thermal sensitivity in polycrystalline MT(XO_4_)_2_ compounds nor UC characterizations of single crystals[32,33] have offered a quantitative assessment on UC efficiency.

On the other hand, two of the previously cited works, NaY(WO_4_)_2_ [27] and NaY(MoO_4_)_2_ [29] deal with micrometric sized materials, while in a third, AgLa(MoO_4_)_2_,[28] the morphology is unknown. Only in NaGd(MoO_4_)_2_,[30] 50x50 nm^2^ square sheets have been studied. These works show a significant spread of the reported *S* values which could be associated to the difference in material composition or to different experimental characterization techniques used in the involved laboratories.

Our purpose in the present work is to present a significant evaluation of the green UC efficiency for eight different DMo/DW hosts in direct comparison to the UC efficiency of Yb:Er:β-NaYF_4_ with the same Yb and Er ion densities, as well as to determine the Yb and Er composition that optimizes the green UC efficiency in these hosts. We address this point by measuring the intensity of the output green photon flux at constant solid angle as well as by simulation of subcutaneous testing using *ex-vivo* breast chicken tissue and both types of compounds (DMo/DW and β-NaYF_4_). Moreover, although there are previous reports of sol-gel synthesis of MT(XO_4_)_2_ compounds, [28,34,35] in this work we present the optimization of the method to avoid particle sintering. Since the reduction of the UC efficiency with particle size is a hot issue for applications, we evaluate the UC efficiency of sub-100 nm UCNPs and we explore their suitability as probes for biomedical applications. For the latter purpose, quasi-spherical sol-gel synthesized Yb:Er:NaLu(MoO_4_)_2_ NPs with diameter ranging 50–80 nm have been perfused in a mouse and their body distribution monitored with lifetime and multiphoton optical microscopies.

We confirm that the thermometric properties of Yb:Er:DMo/DW compounds are superior to those of Yb:Er:β-NaYF_4_. It is also shown that the green UC efficiency of Yb:Er doped Na-based DMo/DW is comparable to that found for Yb:Er:β-NaYF_4_, and that the UC efficiency reduction in the sol-gel synthesized UCNPs decreases only by a moderate factor of 10. This high UC efficiency allowed monitoring the sub-100 nm UCNPs in different body organs, including brain, of a perfused mouse. Finally, a thermal assisted energy transfer excitation mechanism is proposed as responsible for the extraordinary UC properties of DMo/DW compounds.

## 2. Experimental

Comparisons of properties of different materials, either Yb:Er:DMo/DW or Yb:Er:β-NaYF_4_, have been made on the basis of micrometric sized powders prepared by solid state reaction and by hydrothermal (HT) synthesis. The sol-gel synthesis is exclusively used for the preparation of the DMo/DW UCNPs which are eventually perfused in a mouse.

### 2.1 Solid state synthesis

Na_2_CO_3_ (99.5%, Alfa Aesar), WO_3_ (99.8%, Alfa Aesar), MoO_3_ (99.5%, Alfa Aesar) and corresponding Y_2_O_3_ or Ln_2_O_3_ (99.99%, acquired through Shangai Zimei International Co LTD) reagents were used for solid state reaction synthesis of DMo/DW compounds. The homogenized stoichiometric mixtures were firstly heated in air at 650°C (DMo) or 750°C (DW) for 18–24 h, cooled down to room temperature (RT), ground, and then heated at increasing temperatures up to 790°C (DMo) or 885°C (DW). The last thermal annealing to promote crystallization typically lasted 24–48 h.

### 2.2 Hydrothermal synthesis

The HT synthesis of Yb:Er:NaT(XO_4_)_2_ has been previously described in detail.[36] Na_2_MoO_4_·2H_2_O (99.5%, Sigma Aldrich) or Na_2_WO_4_·2H_2_O (99%, Strem Chemicals) and the above indicated Y_2_O_3_ or Ln_2_O_3_ have been used as reagents. Briefly, Y or Ln nitrates were firstly prepared by dissolving together the required stoichiometric amounts of corresponding Y_2_O_3_ or Ln_2_O_3_ sesquioxides in a solution of nitric acid (10 ml of distilled water and 10 ml of 69% HNO_3_), which was heated until complete dryness. Then the Ln^3+^-nitrates were dissolved in 10 ml of distilled water, and a transparent solution of Na_2_(Mo/W)O_4_·2H_2_O with the stoichiometric amount of Mo/W in 10 ml of water was added. After 10 min of stirring the white suspension was transferred to a Teflon-lined autoclave, which was sealed and heated to 170°C for 24 h. The product resulting from the HT reaction was collected by centrifugation, washed several times with distilled water, and dried overnight at 125°C.

Yb:Er:β-NaYF_4_ (7 mmol) samples (7.5, 10 and 20 at% Yb, and for each Yb concentration with 0.25, 0.5, 1, 2.5 and 5 at% Er) were synthesized by adding a previously prepared solution of Ln-nitrates to a clear ethanolic solution (10 ml of ethanol, Emplura Merck, and 20 ml of distilled water) of NaF (99%, Alfa-Ventron), NaF:Ln = 4:1. The formed turbid suspension was stirred and its pH was adjusted to 3 by adding HF (48%, Merck), and then treated at 220°C for 36 h in a Teflon-lined sealed autoclave. The obtained product was in each case collected by centrifugation and washed with ethanol several times, dried overnight at 125°C, and afterwards subjected to 2 h annealing at 300°C to remove defects associated to wet low-temperature synthesis methods, and to promote better crystallization.

### 2.3 Sol-gel synthesis

DMo/DW NPs were obtained by sol-gel following the modified Pechini method. Briefly, Na_2_CO_3_, Y_2_O_3_, Ln_2_O_3_ reagents before described and either (NH_4_)_6_Mo_7_O_24_·4H_2_O (Fluka) or (NH_4_)_6_H_2_W_12_O_40_·nH_2_O (Alfa Aesar) were used as sources for the different required cations. The required amounts of Y_2_O_3_ or Ln_2_O_3_ raw materials were dissolved in 69% HNO_3_ and a suitable volume of deionized water under vigorous stirring and heating at 90°C. When the solution was transparent, Na_2_CO_3_, ammonium molybdate/tungstate, citric acid as complexing agent (CA) and ethyleneglycol (ET) were added to the solution. The molar ratio of chelated metal cations to the CA was 1:4 and the molar ratio of CA to ET was 1:10. The above mixture was stirred for 2 h at 80°C to get a stable precursor solution. Finally, some ammonia was added to adjust the pH value around 4–5. This solution was dried by slow heating at 120°C to obtain a black powder. NPs were obtained by calcination of this powder at 600–800°C temperature range. Higher (> 800°C) calcination temperatures lead to the formation of the Ln_2_O_3_ compounds, likely due to Na and Mo/W evaporation.[37] In order to disperse the obtained NPs the calcined products were mixed with distilled water and treated with a Hielscher Ultrasound Technologies processor, model UP200S operated at 70% of amplitude and 60% of duty cycle. More details of the preparation and characterization methods of NPs can be found in the Supplementary information (SI), in particular a flow chart of the used sol-gel procedures is given in S1 Fig.

### 2.4 Crystallographic characterization

The phase and crystalline quality of the synthesized products was assessed by room temperature (RT) powder X-ray diffraction (pXRD). θ-2θ scans were performed on a Bruker AXS D8 Advance equipment under Cu K_α_ radiation, and the Bragg diffracted light was collected by a Lynxeye detector. The crystalline domain size (d) was determined by the Scherrer formula, d = 0.9λ/Δ2θcosθ (0.9 is the shape factor for spherical domains, λ is the wavelength of the used radiation, and Δ2θ is the full width at half maximum intensity of the analyzed reflection at 2θ angle). For Yb:Er:NaLu(MoO_4_)_2_ we used the 112 Bragg reflection at 2θ≈ 28.5 deg.

Particle morphology and its crystallinity were studied by transmission electron microscopy (TEM) and high resolution transmission electron microscopy (HRTEM) analyses carried out by using a 200 kV JEOL JEM 2100 and a 300 kV JEOL JEM3000F microscopes, respectively.

### 2.5 Dynamic light scattering measurement

The hydrodynamic size of the species present in the dispersions of sol-gel products were determined by dynamic light scattering (DLS) measurements with a Cordouan Technologies particle size analyzer, model Vasco 2.

### 2.6 Optical and spectroscopic characterization

UC was excited at RT with the emission of a 25 A LIMO diode laser (DL). In order to minimize heating of the tested sample due to light absorption, the DL was operated in pulsed mode with a 10% duty cycle and the average current was limited to values lower than 7 A (optical average power <130 mW). Under these conditions the DL output is centered at λ = 973 nm (FWHM≈ 3 nm). The laser beam, delivered through an optical fiber, was focused by a f = 100 mm lens to a spot with ≈ 2 mm of diameter incident onto the sample at 45 deg. The average irradiation density was ≤ 4 W/cm^2^. For UC emission evaluation the fluorescence was collected by a condenser lens (50 mm of diameter) parallel to the sample surface and focused to the entrance of a fiber ended Spectral Products compact spectrometer, model SM440. The residual excitation light was removed from the detection system by an Edmund Optics PO:7MOD-11081 short-wavelength pass filter with a transmission band between 402 nm and 838 nm. All spectra were corrected by the spectrometer background and its spectral response. The powdered samples were pressed and leveled to a reference horizontal plane and all UC measurements were collected under the same geometry to allow the evaluation of the relative efficiency. The UC efficiency was evaluated from the area under the green UC emissions. Standard optical absorption spectra were acquired with Varian, Cary 500, spectrophotometer.

### 2.7 Techniques for nanoparticle distribution in a mouse model

Adult mice were deeply anesthetized with CO_2_ and perfused transcardially first with a solution containing NPs followed by 4% paraformaldehyde in phosphate buffer saline (PBS, pH 7.4) solution as described before.[38] Several organs were collected and further fixed in the same fixative for a 24 h period at 4°C. Mouse organs were kept in PBS at 4°C until further analysis. All these procedures with animals complied the Spanish and European legislation on the protection of animals used in research, and the protocols were approved by the CSIC Ethics Committee on Animal Experimentation.

### 2.8 Fluorescence lifetime imaging (FLIM) and multiphoton microscopies

For FLIM we used an Alba laser-scanning module (ISS, Inc., Champaign, IL, USA) interfaced with an inverted Nikon Eclipse T*i* microscope. Fluorescence was excited through a Nikon S Fluor 40× /0.9 NA DIC M/N2 dry objective. For excitation we used a Ti:Sapphire tunable Spectra Physics laser (model Mai Tai DeepSee, delivering laser pulses with 100 fs of pulse duration at a repetition rate of 80 MHz) coupled to an acousto-optic pulse picking modulator. NPs were excited at λ = 973 nm with a laser power of 1 nW at the sample and the emission was selected through a λ_0_ = 530±43 nm bandpass filter. Tissue images were acquired exciting at λ = 900 nm with a laser power of 450 nW at the sample, and the emission was selected through a λ_0_ = 445±20 nm bandpass filter. With a scan area of 256×256 pixels and a pixel dwell time of 32 μs, we could image a 160×160 μm^2^ area in sample at 625 nm pixel size and 0.625×0.625×1.000 μm^3^ (x,y,z) voxel size.

FLIM was performed following the digital frequency domain method and using a FLIM card previously described,[39] which was developed at the Laboratory of Fluorescence Dynamics (University of California, Irvine, CA) and implemented in the Alba module. Data were acquired and processed according to the phasor FLIM method implemented in the VistaVision software (ISS, Inc., Champaign, IL, USA).[40] Details about the phasor FLIM method can be found in previous literature [41,42] and it is briefly described in the SI. A concentrated fluorescein at pH 9.0 was used as fluorescence lifetime standard and measured before each sample for fluorescence lifetime calibration. The fluorescein fluorescence lifetime (τ = 4.04 ns) was determined independently using a fluorometer (PC1 by ISS, Inc., Champaign, IL, USA).

Tiled 2-color multiphoton images were obtained on an upright LSM 780 Zeiss confocal microscope coupled to a Ti:Sapphire tunable Spectra Physics laser (model Mai Tai DeepSee, providing laser pulses with 80 fs of pulse duration at a repetition rate of 80 MHz) and equipped with a deep in Plan Apochromat 20x /1 NA DIC 0,17 M27 75 mm water immersion objective. NPs were excited at λ = 973 nm with an average laser power of 8 nW at the sample, and the emission was collected through a spectral GaAsP photodetector in the λ = 517–562 nm range. Tissues were excited at λ = 850 nm with laser power of 600 nW at the sample, and the emission was collected by a photomultiplier tube in the λ = 600–650 nm range. Images of 2867×2867 pixels at a pixel dwell time of 1.52 μs were obtained by 3x3 tile scans to document sample areas of 1190×1190 μm^2^, with a pixel size of 415 nm and a 0.415×0.415×1.000 μm^3^ (x,y,z) voxel size. Three dimensional (3D) rendering was obtained from z-stacks of 38–45 optical sections with 2 μm step size under the same scanning conditions. IMARIS (Bitplane Scientific Software, Zurich, CH) and Fiji software were used for 3D rendering and post-processed composite images.

## 3. Results and discussion

### 3.1 Comparison of UC efficiency of Yb:Er doped DMo/DW versus β-NaYF_4_

**Fig 1:**
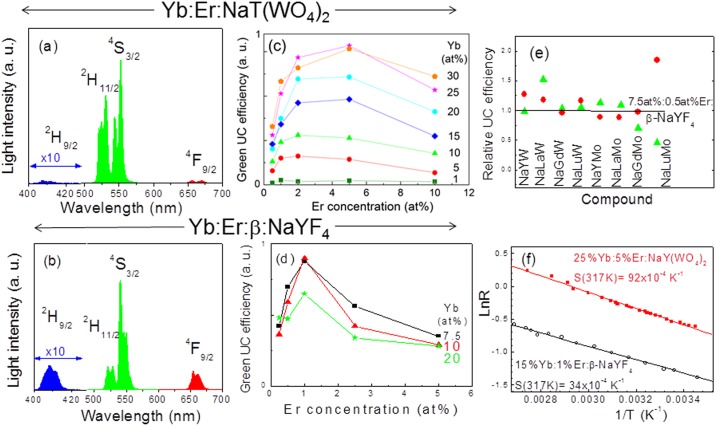
Comparison of up-conversion properties of DMo/DW and β-NaYF_4_. RT UC characteristics of Yb:Er:DMo/DW in comparison to Yb:Er:β-NaYF_4_ upon cw DL excitation with 4 W/cm^2^ of light density. a) UC spectral distribution of 25at%Yb:5at%Er:NaLu(MoO_4_)_2_ synthesized by solid state reaction. (b) UC spectral distribution of HT 10at%Yb:1at%Er:β-NaYF_4_. Indicated multiplets are those emitting to the ground ^4^I_15/2_ one. Note that for a given material the intensity of blue, green and red UC, although arbitrary, is in the same scale. (c) Evolution of the green UC efficiency of Yb:Er:NaY(WO_4_)_2_ as a function of Yb and Er concentrations. (d) Evolution of the green UC efficiency of HT Yb:Er:β-NaYF_4_ as a function of Yb and Er concentrations. (e) Maximum green UC efficiencies achieved for 15at%Yb:1at%Er:NaT(XO_4_)_2_ (shorten as NaTX) synthesized by solid state reaction (●) or HT (▲) methods, relative to HT 7.5at%Yb:0.5at%Er:β-NaYF_4_ (line). (f) Comparison of NaY(WO_4_)_2_ and β-NaYF_4_ thermometric properties.

Fig 1 shows a comparison of the UC emission of Yb:Er:NaT(XO_2_)_4_ and Yb:Er:β-NaYF_4_ compounds. Fig 1a and 1b show the UC emission spectra. Two main green band sets corresponding to Er^3+^ electronic de-excitation from ^2^H_11/2_→^4^I_15/2_ (λ≈ 520–540 nm) and from ^4^S_3/2_→^4^I_15/2_ (λ≈ 540–560 nm) are observed. Additionally, other weaker UC emissions may be observed for the blue ^2^H_9/2_→^4^I_15/2_ (λ≈ 400–420 nm, this band is very weak in DMo/DW compounds) and red ^4^F_9/2_→^4^I_15/2_ (λ≈ 630–700 nm) Er^3+^ transitions. It is worth noting that the relative intensity of the latter red UC emission is much lower in NaT(XO_2_)_4_ compounds than in β-NaYF_4_ one. This feature extends to all host compositions of the DMo/DW compounds studied and for any Yb and Er concentrations. Other notorious difference between the green UC spectrum in Yb:Er:DMo/DW and Yb:Er:β-NaYF_4_ is that the ^2^H_11/2_/^4^S_3/2_ UC intensity ratio is much larger in the formers.

The efficiency of non radiative resonant transfer lies on donor-acceptor distance, thus in order to compare the UC efficiency of DMo/DW compounds with that of β-NaYF_4_ reference compound, similar Ln densities (in at/cm^3^) must be considered. For this purpose it must be taken into account that the tetragonal unit cell of DMo/DW, with a volume V≈ 300 Å^3^, contains two formula units (Z = 2),[43] while the unit cell of the hexagonal β-NaYF_4_ fluoride (formulated as Na_1.5_Y_1.5_F_6_, V = 108.99 Å^3^), contains only one formula unit (Z = 1).[44] Thus similar Ln densities (therefore equivalent average Ln-Ln distance) imply double atomic percent substitution in the tetragonal scheelite host than in the hexagonal gagarinite one. Moreover, the optimum Yb:Er substitution ratios can be different between both hosts due to the specific details of the energy migration. In order to resolve these points Fig 1c and 1d show a comparison of the green UC efficiency for several Yb:Er ratios in NaY(WO_4_)_2_ and β-NaYF_4_ hosts, respectively. The optimum dopant concentration for maximum Yb:Er:β-NaYF_4_ UC efficiency occurs for about 10at%Yb:1at%Er, while maximum UC efficiency in NaY(WO_4_)_2_ occurs at 25at%Yb:5at%Er substitution, i.e. shorter Ln-Ln distance is required in the scheelite host, likely associated to a lower host energy migration length.

In order to determine the most favorable DMo/DW host for Yb:Er green UC Fig 1e shows a comprehensive comparison of eight DMo/DW compounds doped with 15at%Yb:1at%Er and synthesized either by hydrothermal or solid state reaction methods. The products were repeatedly annealed in order to enlarge the crystalline domains leading to improvements in the UC efficiency. It can be observed that for these micrometer sized compounds the UC efficiency depends little on the DMo/DW host and on the synthesis method, and the obtained green UC efficiencies are equal and even larger than that found for HT synthesized 7.5at%Yb:0.5at%Er:β-NaYF_4_, taken as reference.

**Fig 2:**
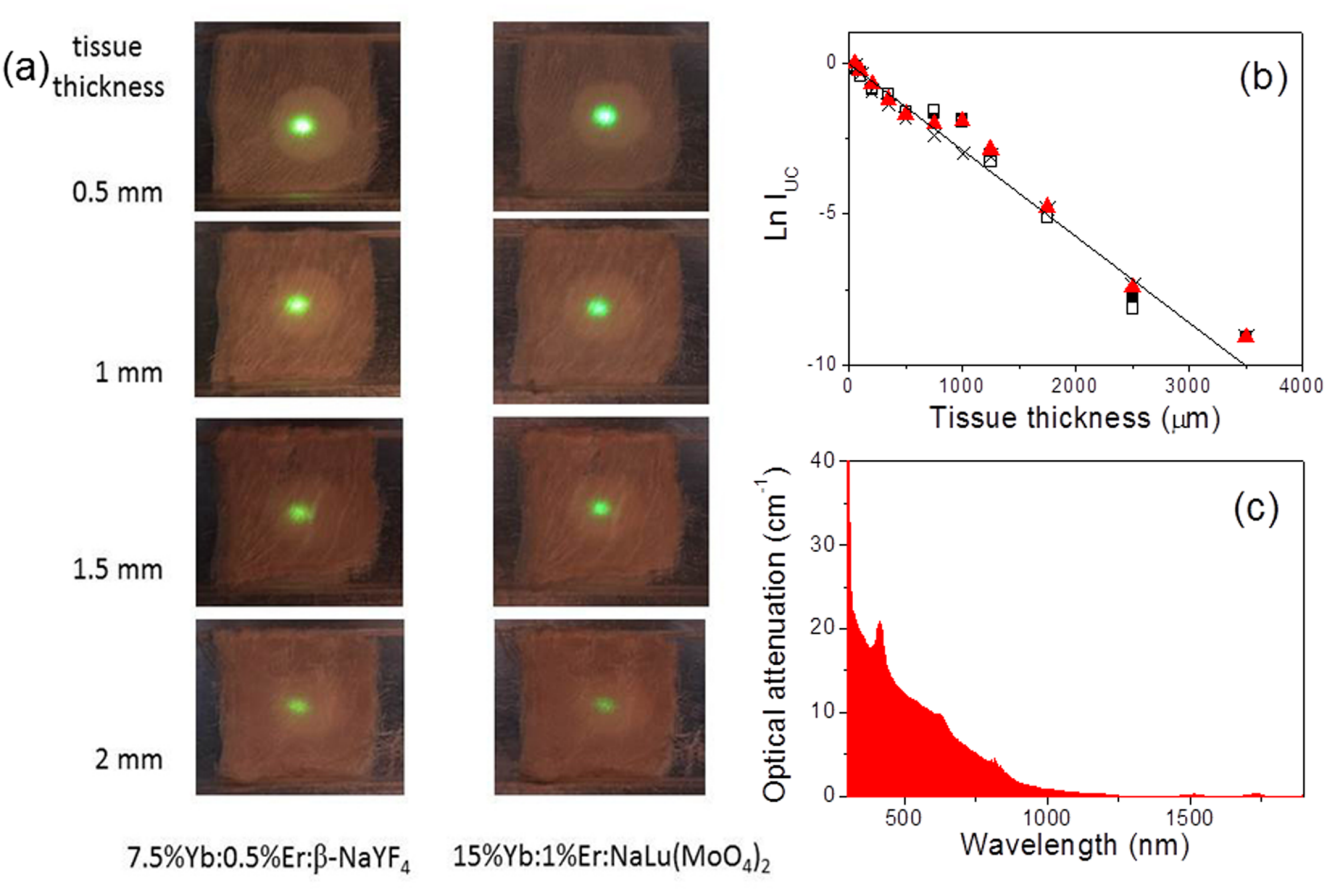
Simulation of subcutaneous up-conversion detection. (a) Images of the UC emission of the reference 7.5at%Yb:0.5%Er:β-NaYF_4_ and of 15%Yb:1%Er:NaLu(MoO_4_)_2_ compounds lying below several tissue thicknesses of *ex-vivo* chicken breast. The images were continuously excited with 3 W/cm^2^ of light density. (b) Semi-logaritmic representation of the green UC intensity as a function of the thickness of the *ex-vivo* chicken breast tissue tested with different UC sources: 7.5at%Yb:0.5at%Er:β-NaYF_4_ (HT, ■), 25at%Yb:5at%Er:NaY(WO_4_)_2_ (solid state reaction, □), 15at%Yb:1at%Er:NaLu(MoO_4_)_2_ (solid state reaction, ×), and 15at%Yb:1at%Er:NaLa(WO_4_)_2_ (HT, ▲). (c) Optical attenuation of *ex-vivo* chicken breast tissue.

As a further macroscopic evidence of the equivalence of UC efficiency in Yb:Er:β-NaYF_4_ and Yb:Er:DMo/DW compounds and as a proof of concept of its application for subcutaneous testing, we have monitored the UC attenuation in an *ex-vivo* chicken breast, Fig 2 summarizes these results. For this purpose, tissue sections with individual thickness of 50 μm were sliced with a microtome and piled-up to obtain different tissue thicknesses. Fig 2a shows the results upon cw NIR irradiation with the DL. It is worth noting that with both materials a tissue thickness in excess of 2 mm could be efficiently penetrated by the DL light and UC emission still detected with conventional methods (a Canon EOS 600D camera was used for recording).

Fig 2b shows a quantitative evaluation of the UC intensity attenuation for increasing tissue thickness (t). Considering an exponential attenuation law (*I* = *I*_0_*e*^−κ*t*^), the attenuation coefficient results κ = 27 cm^-1^, independently of the DMo/DW or β-NaYF_4_ compound used as UC source. It is obvious that the determined κ value is much larger than the optical absorption coefficient of the breast chicken tissue at green and IR wavelengths, typically α = 0.4–0.8 cm^-1^ and α< 0.2 cm^-1^, respectively.[45] In fact, Fig 2c shows that light attenuation of the *ex-vivo* chicken breast tissue is composed of an increasing (with decreasing wavelength) background and some absorption peaks at specific wavelengths. The background is related to light scattering, therefore the UC attenuation observed in Fig 2b is basically related to the defocusing of the excitation beam upon propagation through the tissue, thus reducing the light density at the focus. This has a deep influence of the overall photonic process since the UC intensity (I_UC_) is non-linearly related to the excitation pumping intensity, I_P_, i.e. I_UC_≈ I_P_^n^, n being typically equal to the number of photons involved in the UC mechanism (n = 1.5–3).

### 3.2 Ratiometric UC thermometry

Nanothermometry is a remarkable application of UCNPs, which is related to the Boltzmann electron population redistribution between the close in energy ^2^H_11/2_ and ^4^S_3/2_ Er^3+^ multiplets. Briefly, the change with temperature of the *R* = I(^2^H_11/2_)/I(^4^S_3/2_) UC intensity ratio determines the material UC ratiometric thermal sensitivity, *S = dR/dT*. More explicitly, *R* = *C*×exp(-Δ*E*/*k*_*B*_*T*) and *S* = *R*×(Δ*E*/*k*_*B*_*T*^2^), where *k*_*B*_ is the Boltzmann constant and Δ*E* is the energy gap between ^2^H_11/2_ and ^4^S_3/2_ multiplets. *C* and *ΔE* are characteristics of each considered material and are usually calculated from Ln*R* vs 1/*T* representations. Fig 1f shows a comparison of such representation for 25at%Yb:5at%Er:NaY(WO_4_)_2_ and 15at%Yb:1at%Er:β-NaYF_4_. The UC ratiometric thermal sensitivity obtained at *T* = 317 K (44°C) are *S* = 92×10^−4^ K^-1^ and *S* = 34×10^−4^ K^-1^, respectively. The high *S* value of the former is similar to those reported for other DMo/DW compounds.[27–29] This confirms that the UC ratiometric thermal sensitivity of Yb:Er-doped DMo/DW hosts is unless 3 times larger than that of the reference fluoride. Using the results shown in Fig 1f, the thermal resolution, *r* = *σ*_*R*_/*S* (*σ*_*R*_ is the standard deviation of *R*) are 0.7 K and 1.8 K for the DW and β-NaYF_4_, respectively. Although thermal resolution is not a material property and precautions must be taken when comparing data obtained with different experimental setups, the present results show the advantage of using the DMo/DW instead of the reference fluoride for thermal measurements.

Nevertheless, it must be noted that thermometric measurements are complex due to several uncertainties. On the one hand, the considered DMo/DW materials have low thermal conductivity (1.47 and 1.62 Wm^-1^K^-1^ at RT for *a* and *c* directions, respectively)[46] and the excitation laser beam may cause light induced heating of the powder, thus thermal gradients from the heating element to the powder surface as well as radial ones associated to the Gaussian character of the excitation laser beam are present. On the other hand, material-related uncertainty may potentially arise from composition, crystallinity and morphologic variations associated to synthesis processes.

In order to estimate a realistic uncertainty for the thermal parameters determined in this work we have conducted several additional experiments whose results are included in the SI. First, a 25at%Yb:5at%Er:NaY(WO_4_)_2_ sample prepared by solid state reaction undergone three sequential heating/cooling round trip cycles, and *S* was evaluated independently for each one of the six up or down trips and for the whole data set, see S7 Fig. In that case the average sensitivity obtained was *S*(317K) = 99×10^−4^ K^-1^ with an standard deviation (calculated from the individual cycles) of *σ*_*S*_(317 K) = 4.6×10^−4^ K^-1^ and the thermal resolution taking into account the whole data set was *r* = 0.9 K. Alternatively, when the resolution is calculated from ten measurements taken at the nominal constant temperature of 317 K its value is reduced to *r* = 0.65 K, what shows the importance of the calibration procedures for resolution calculation.

Moreover, measurements were made with the sensing thermocouple buried at different distances (h = 1.25 mm, 1.9 mm and 2.4 mm) from the heating element in the powder of a new 25at%Yb:5at%Er:NaY(WO_4_)_2_ sample prepared also by solid state reaction under extensive sequential heating at 850°C for 60 h and at 880°C for 3 h, see S8 Fig. It is observed that *S* decreases by less than 5% with increasing distance between the resistive heating element and the thermocouple union. However, it is worth noting that the sensitivity obtained for the latter sample with extensive thermal annealing is about 10% lower than that obtained for the previous preparation despite the same nominal sample composition. This shows the importance of material synthesis control for the DMo/DW considered which are prone to evaporate Na and Mo at high temperature (>800°C)[37] leading to traces of Na_2_(Mo/W)O_4_ and Ln_2_O_3_ phases in overheated polycrystalline materials.

### 3.3 Sol-gel synthesis of Yb:Er:NaLu(XO_4_)_2_ nanoparticles

DMo/DW NPs with optimum composition for green UC emission, i.e. 25at%Yb:5at%Er:NaLu(MoO_4_)_2_ and 25at%Yb:5at%Er:NaLu(WO_4_)_2_, see Fig 1c, have been prepared by the sol-gel method. Lu compositions were selected because they are the members with lowest melting temperature for all Y and Ln series,[47] which is expected to easy the phase synthesis at low temperature, thus minimizing particle sintering.

The precursor black powders obtained by sol-gel were calcined to different temperatures (600, 650, 700, 750 and 800°C) and times (2, 3, 4, 6, 8, 10 and 12 h). The minimum calcination temperature (550°C) was determined by the need of full elimination of carbonaceous compounds (see S2 Fig). Sol-gel NaLu(WO_4_)_2_-intended products synthesized at short calcination times (< 6h) and low temperatures (≤ 750°C) show the presence of residual amounts of the Na_2_W_2_O_7_ phase (see S3b Fig). This undesired phase is completely removed by increasing calcination time and temperature. This problem does not exist in the sol-gel preparation of NaLu(MoO_4_)_2_ compounds, for which the scheelite phase is exclusively observed even after only 2 h of calcination at 600°C.

**Fig 3:**
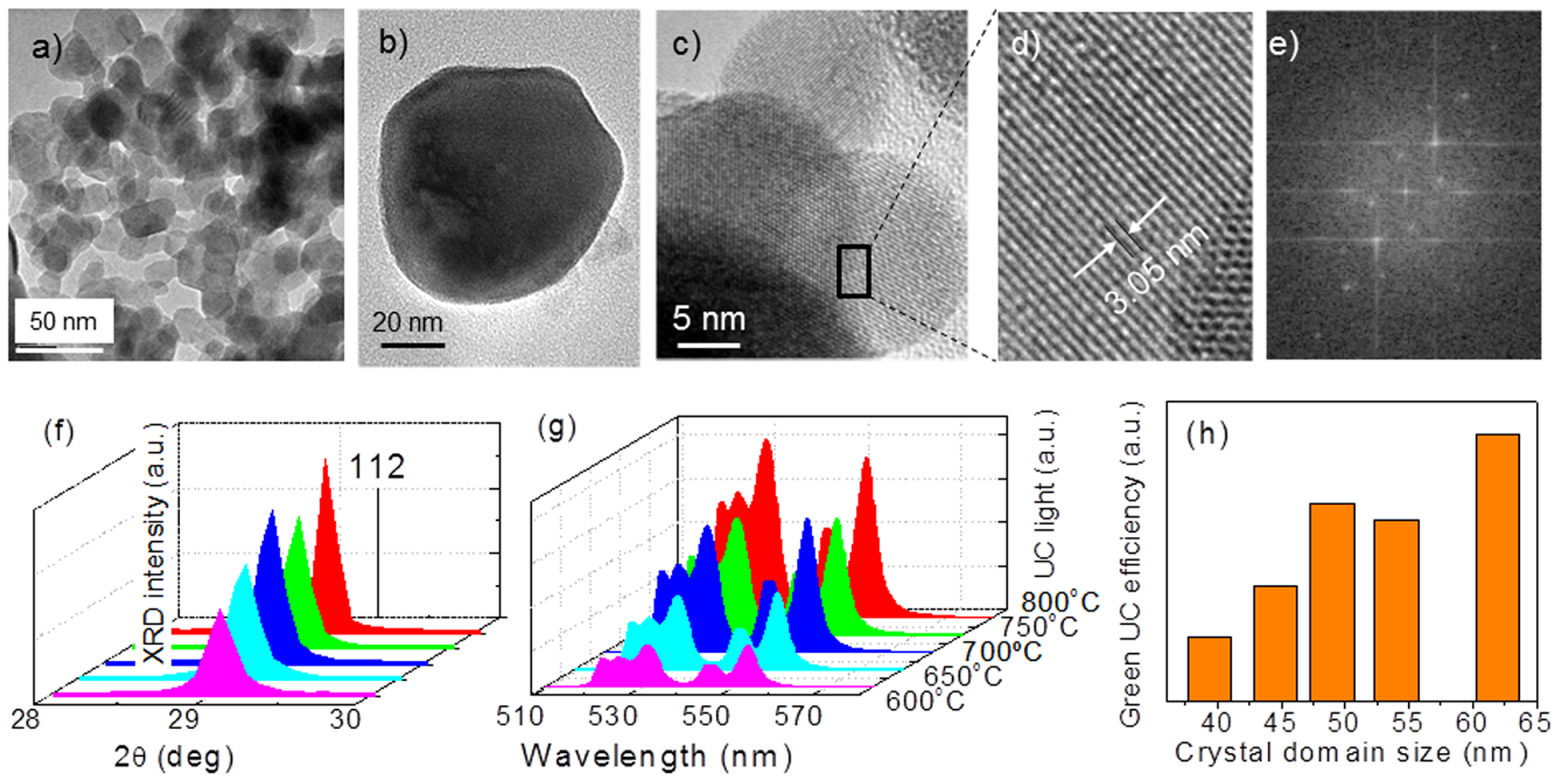
Characterization of sol-gel nanoparticles. Properties of sol-gel synthesized Yb:Er:NaLu(XO_4_)_2_ UCNPs. (a,b) TEM images of 25at%Yb:1at%Er:NaLu(MoO_4_)_2_ sol-gel products after 4h calcination at 600°C. (c) HRTEM image of highly crystalline 15at%Yb:1at%Er:NaLu(WO_4_)_2_ rounded NPs with ≈20 nm of diameter. (d) Selected area of the HRTEM image. Observed lattice fringe distances are 0.305 nm, which matches the (112) interplanar spacing of NaLu(WO_4_)_2_. (e) Fast Fourier transform of the above image showing particle crystallinity. (f) pXRD scans of 25at%Yb:5at%Er:NaLu(MoO_4_)_2_ sol-gel NPs as a function of calcination at increasing temperature for a 12 h period. (g) Evolution of the green UC intensity of 25at%Yb:5at%Er:NaLu(MoO_4_)_2_ sol-gel NPs as a function of calcination at increasing temperature for a 12 h period. (h) Relationship between green UC efficiency of 25at%Yb:5at%Er:NaLu(MoO_4_)_2_ sol-gel NPs and their crystalline domain size.

Fig 3 shows a morphological characterization of the obtained NPs. Fig 3a–3e show characteristic TEM and HRTEM images of 25at%Yb:5at%Er:NaLu(MoO_4_)_2_ sol-gel products after 4 h calcination at 600°C. At the lowest studied temperature (600°C) and for short calcination time (4 h) numerous quasi-spherical NPs with diameter in the 50–80 nm range appear detached or superimposed. For longer calcination times, 12 h, at the same temperature the NPs show a tendency to cluster but still their individual character is preserved. At higher calcination temperature, 700°C, some detached particles are observed even though their average size grows to 150–250 nm range, but often the particles are sintered forming rows. Grain boundaries between different particles are easily seen at this stage. Calcination at 800°C leads to general particle sintering forming superstructures with micrometric size. As indicated in Fig 3f–3h, this sintering process is followed by a growth of the crystal domain size and by an improvement of the UC efficiency, thus it should be concluded that the use of low calcination temperature (600°C) and short annealing time (4–12 h) is required for the production of isolated NPs, which in turn makes NaT(MoO_4_)_2_ compounds preferred over NaT(WO_4_)_2_ counterparts due to the better phase purity upon 600°C calcination. A more general view of the effect of calcination temperature and time on the particle morphology is presented in the SI. As this point, it is worth to note that Yb:Er:NaLu(MoO_4_)_2_ NPs calcined at 600°C have a crystalline domain size of ≈40 nm, which is compatible with the HRTEM results described above.

**Fig 4:**
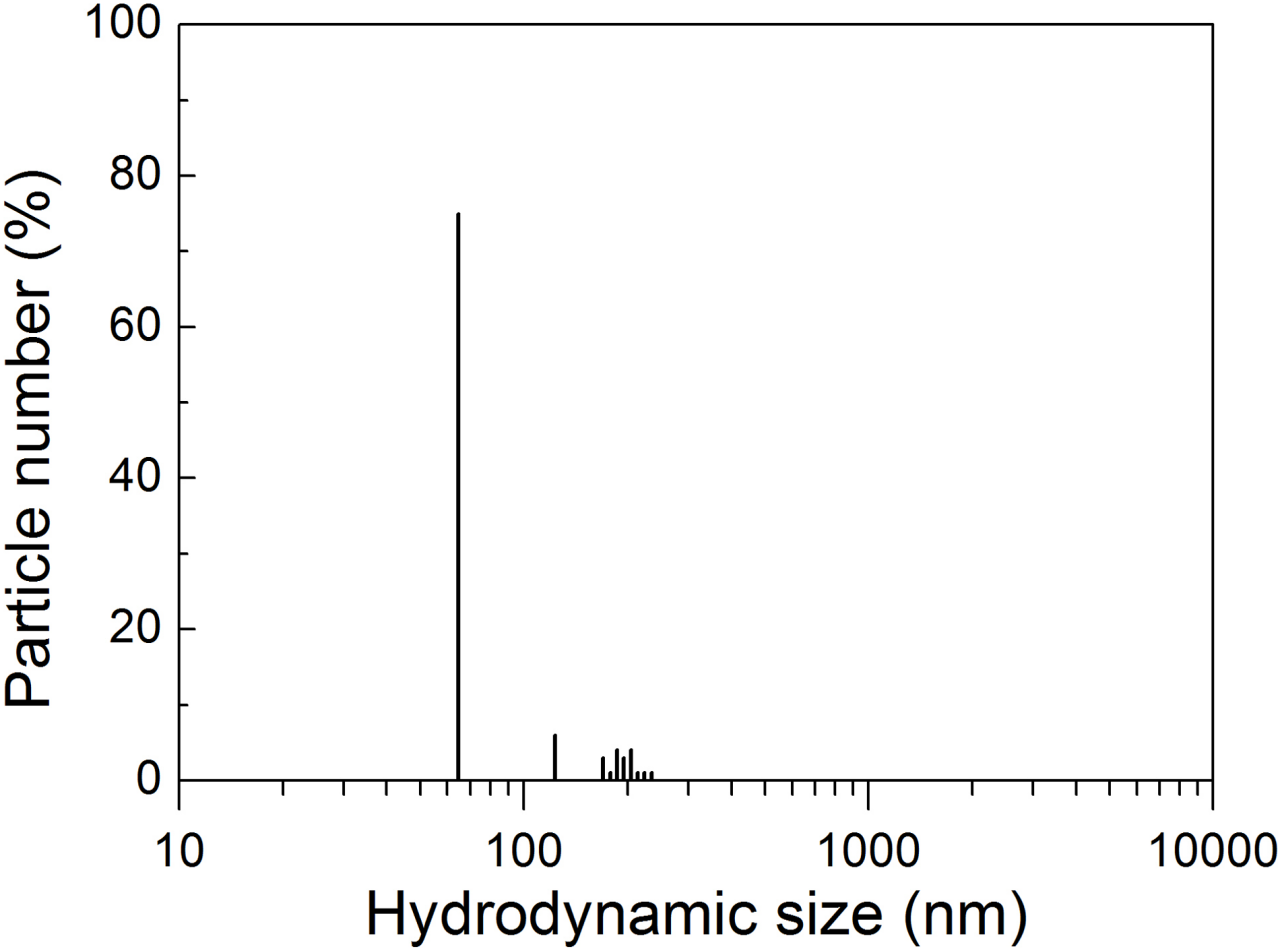
DSL characterization of NP size. Hydrodynamic particle size distribution in water of 25at%Yb:5at%Er:NaLu(MoO_4_)_2_ sol-gel products obtained after 6h of 600°C calcination and 12 min of processing with ultrasounds.

To further confirm the water dispersibility of the NPs we have studied by DLS the size distribution of the species present in dispersions of the 600°C calcined 25at%Yb:5at%Er:NaLu(MoO_4_)_2_ sol-gel products additionally processed with ultrasounds. Fig 4 shows an example of the hydrodynamic size distribution of the above products treated 12 min in the ultrasound processor. In this case 75% of the total species found corresponds to free NPs with 65 nm size with a polydispersity index PDI = 0.1745. The rest are presumable clusters of non sintered NPs that could be fully removed by longer ultrasound processing. Full details of the DLS study are given in the SI. The general picture drawn from the DLS study fully agree the above TEM results: NPs present in the dry powder obtained by 600°C calcination are clustered, but these clusters can be broken by ultrasound processing releasing free and water dispersible NPs with size below 100 nm.

Based on the comparison of the UC output intensity, the green UC efficiency of sol-gel synthesized 25at%Yb:5at%Er:NaLu(MoO_4_)_2_ NPs with average size 50–80 nm is reduced by a factor ≈10 with regards to micrometer sized products. This reduction is similar to that observed for 20at%Yb:2at%Er:β-NaYF_4_ NPs with size ≈ 100 nm.[25] The UC ratiometric thermal sensitivity at 317 K of these 25at%Yb:5at%Er:NaLu(MoO_4_)_2_ NPs has been determined as *S* = 127×10^−4^ K^-1^, see S9 Fig, i.e. ≈ 3.5 times larger than that obtained for Yb:Er:β-NaYF_4_.

### 3.4 Nanoparticle distribution in mouse body

**Fig 5:**
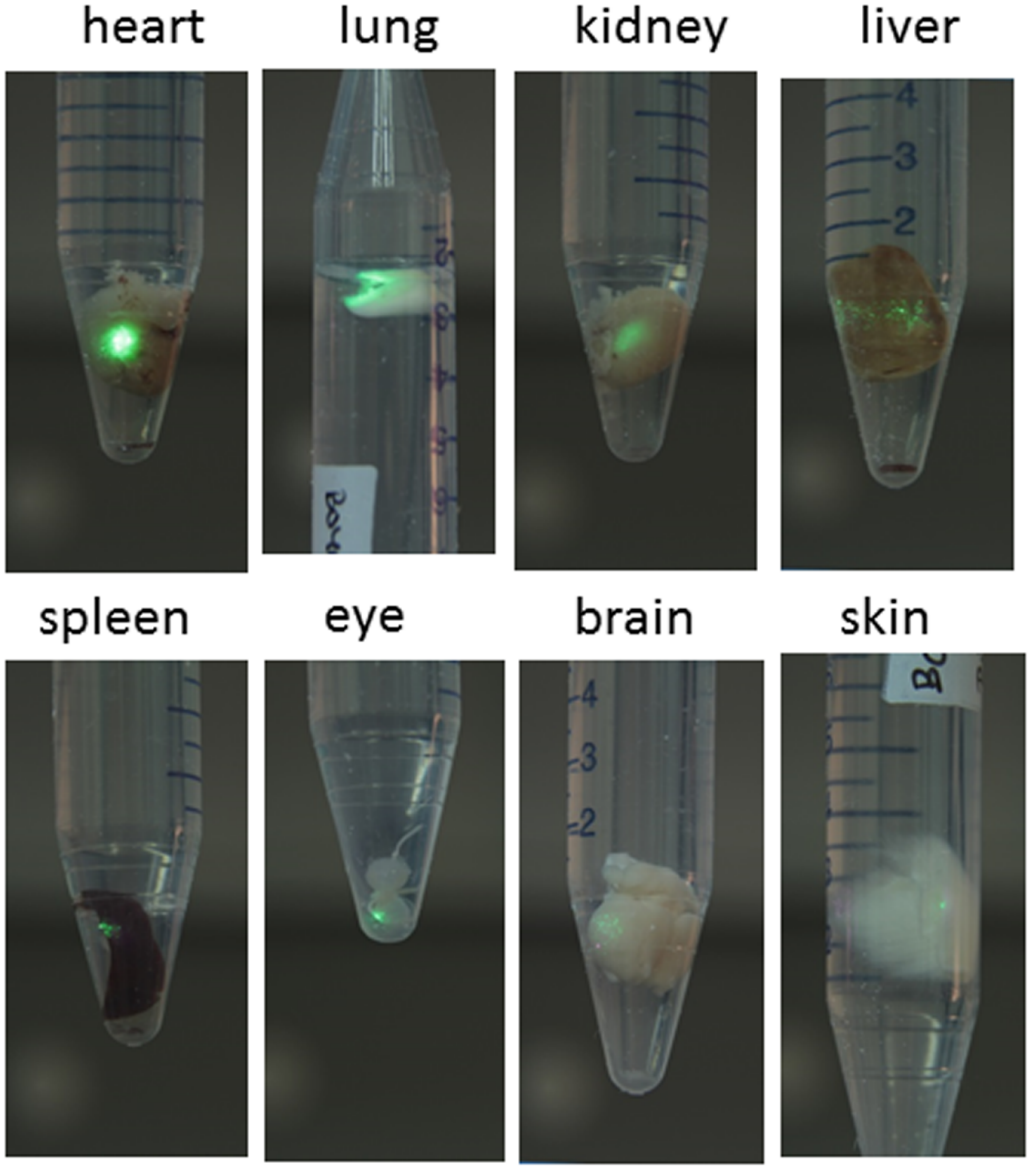
Macroscopic up-conversion characterization of perfused mouse organs. UC emission from organs of a mouse after perfusion with sol-gel synthesized 25at%Yb:5at%Er:NaLu(MoO_4_)_2_ NPs.

In order to explore the potential of the above NPs for body imaging and sensing we perfused the 25at%Yb:5at%Er:NaLu(MoO_4_)_2_ NPs obtained after calcination at 600°C (12 h) in an adult mouse. Afterwards, different mouse organs and tissues were examined. Fig 5 shows the excised whole mouse organs under irradiation with the 973 nm emission of the DL. From this macroscopic view it is obvious that NPs reach all monitored mouse organs and even signals from brain and eye are detected, while the signal from skin is comparatively weaker.

**Fig 6:**
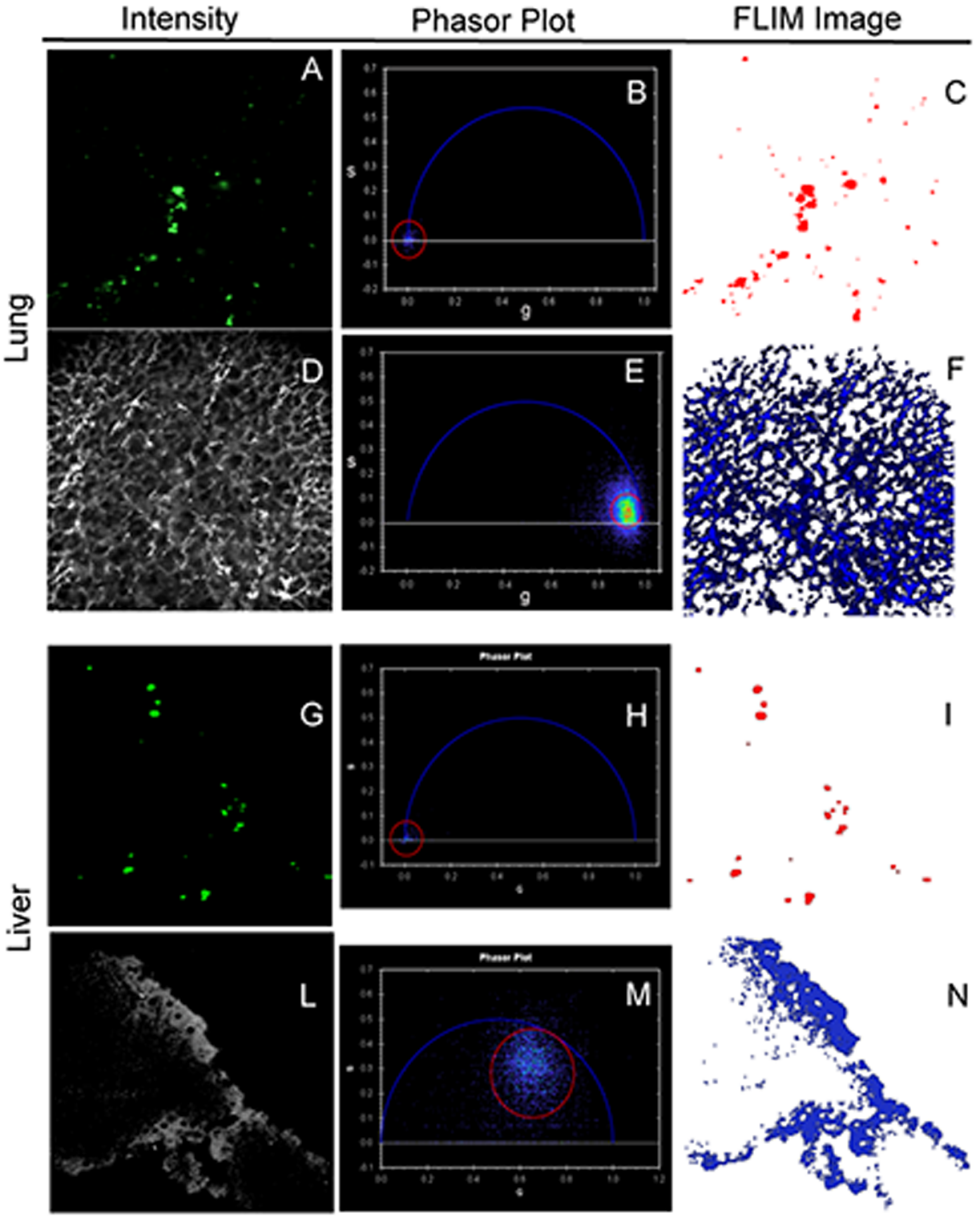
FLIM assessment of up-conversion from mouse perfused sol-gel nanoparticles. Combined fluorescence intensity and FLIM analyses of tissue sections after perfusion with sol-gel synthesized 25at%Yb:5at%Er:NaLu(MoO_4_)_2_ NPs. Intensity images of UCNPs at λ_EXC_ = 973 nm, λ_EMI_ = 530±43 nm (a, g), and corresponding fluorescence lifetime distributions represented by the phasor transform in polar coordinates (b, h). The FLIM images (c, i) show the localization of the pixels enclosed in the red-circled cursor. The lung (d) and liver (l) sections in which the particles are found were imaged at λ_EXC_ = 900 nm and λ_EMI_ = 445±20 nm. The fluorescence lifetime distributions associated to the tissues are represented by the phasors enclosed by the red-circled cursor in (e) and (m). Independently on the source of the tissue signals, SHG (lung) or autofluorescence (liver), UCNPs fluorescence lifetime distributions are clearly distinguishable from that of the tissues in which the particles are trapped. Images are shown in pseudo-color scales.

To determine without doubt the presence of UCNPs in mouse organs we have combined fluorescence microscopy with fluorescence lifetime analysis. In thick organ sections the NPs were detected by their UC fluorescence intensity obtained at λ_EXC_ = 973 nm and λ_EMI_ = 530±43 nm (Fig 6a), and also by their fluorescence lifetime as shown by the phasor distribution centered at (g = 0,s = 0) in the polar plot of Fig 6b. All pixels associated to the fluorescence lifetime distribution enclosed in the red circle of Fig 6b are represented in the FLIM image (Fig 6c). The fluorescence lifetime distribution obtained at λ_EXC_ = 973 nm is concentrated near to (g = 0,s = 0), indicating the presence of long-lived emissions, τ> 10 ns. With our experimental set-up (100 fs laser pulse at repetition rate of 80 MHz equal to 12.5 ns, with pulse picker) we are unable to precisely determine lifetime longer than 10 ns, thus the distribution corresponding to NPs is squeezed at the (g = 0,s = 0) polar coordinates. Nevertheless, the perfect superimposition of FLIM and intensity images clearly identifies the NPs entrapped in the examined lung section. The structure of the lung section analyzed in this example is shown in Fig 6d, and it was captured under λ_EXC_ = 900 nm and λ_EMI_ = 445±20 nm acquisition conditions. Under these conditions, the lifetime distribution close to (g = 1,s = 0) in the polar plot (Fig 6e) indicates the predominance of second harmonic generated (SHG) signal, with lifetime close to zero, rather than tissue autofluorescence.

A different example is provided by the study of a liver section shown in Fig 6g to 6n. UCNPs are again detectable by both fluorescence emission intensity (Fig 6g) and fluorescence lifetime (Fig 6h and 6i) images in the mouse liver, and again the corresponding coordinates in the phasor plot are near (g = 0,s = 0). In contrast to the lung example above, in liver autofluorescence prevails (Fig 6l), as indicated by the short, but no-zero, diffuse phasor distribution (Fig 6m and 6n) in a range of τ = 0.5–2 ns.[42] Similar lifetime values have been typically observed in previous studies of tissue autofluorescence.[48–50]

Taking into account that the emission kinetic of Yb:Er:NaLu(MoO_4_)_2_ compounds is controlled by the Yb^3+^ de-excitation rate with a lifetime in the 50 μs to 1 ms range, depending on Yb concentration and material microstructure,[36] these results rule out autofluorescence or second harmonic contributions to the fluorescence intensity and lifetime images showing UCNPs entrapped in different mouse organs.

**Fig 7:**
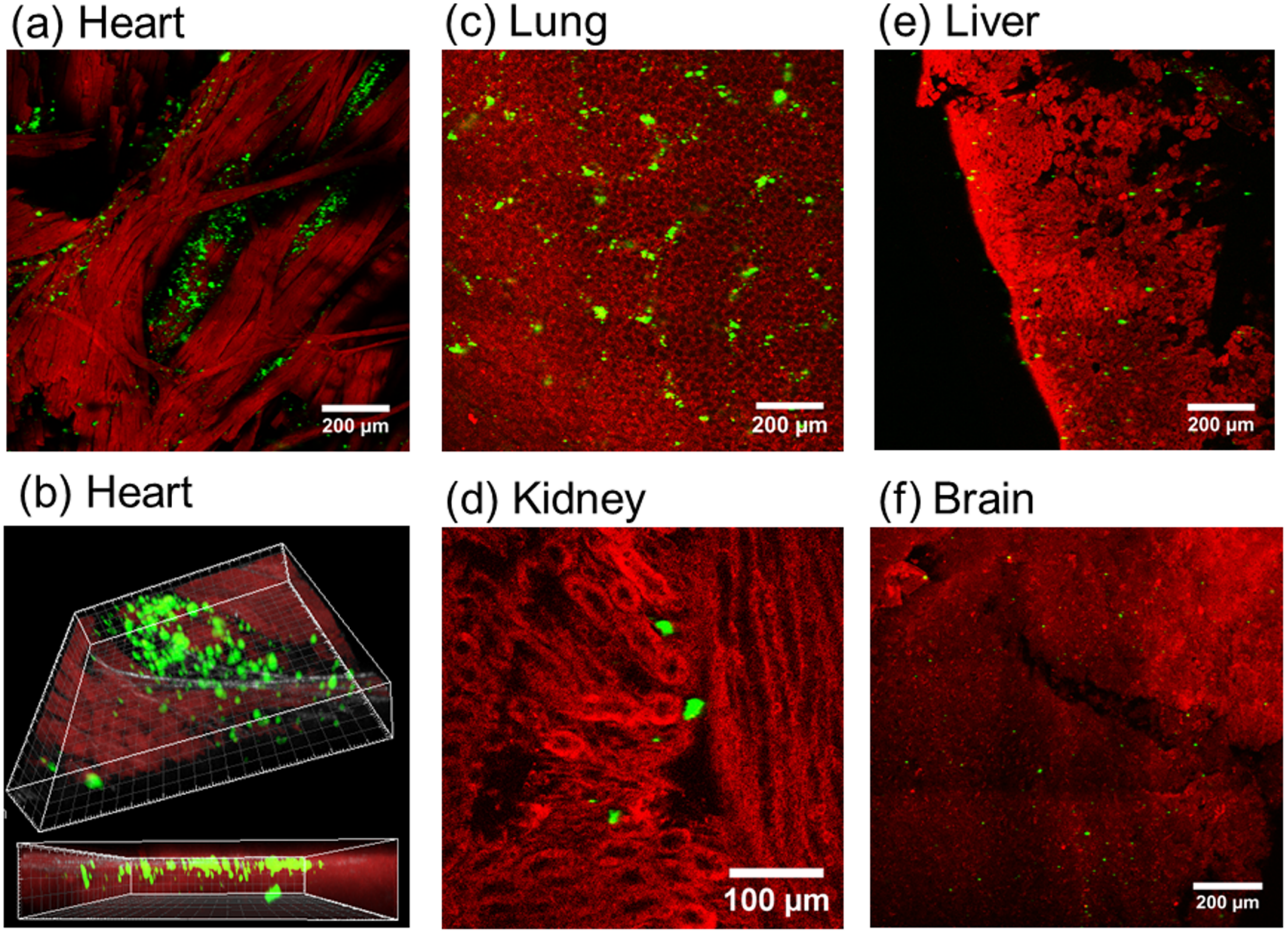
Bi and three dimensional micrographs of sol-gel nanoparticles perfused in mouse organs. Composite images showing tissue autofluorescence (red) and up-conversion of sol-gel synthesized 25at%Yb:5at%Er:NaLu(MoO_4_)_2_ NPs perfused into mouse organs (green). Heart (a,b), lung (c), kidney (d), liver (e) and brain (f). Composite images were obtained from 3x3 tile scans for a total area of 1190 x1190 μm^2^. A 3D rending of a z-stack of 38 planes of one tile scan acquired at 2 μm step size is also shown for the heart sample (b). Images are shown in pseudo-color monochromatic scales.

To document the body distribution of NPs, we have excised five organs from a treated mouse and examined them by mutiphoton microscopy. Fixed organs were divided in halves to expose the interior of the tissue. The internal side of each section was positioned in a bottom glass dish. Fig 7 shows a gallery of composite images tiled over large tissue areas that illustrate the structural features of each examined organ and the distribution of NPs accumulated in the interior.

In the heart, cardiomyocytes can be recognized by the elongated shape with striations similar to those on skeletal muscle cells. (Fig 7a). NP deposits can be observed in the space between these fibers. A 3D reconstruction obtained from a z-scan is also shown in Fig 7b. Fig 7c shows the autofluorescence image of a portion of lung parenchyma depicting pulmonary alveoli, numerous NPs were found throughout the tissue. Fig 7d shows deposits of NPs found in the renal cortex in which numerous cortical glomeruli and associated tubular structures are visible. In Fig 7e, a portion of a liver lobe, partially depicting several hepatic lobules with the central vein most apparent, is observed to contain NPs in each lobule. In Fig 7f, a portion of the brain cortex with undefined morphological structure shows the presence of some NPs in between the nervous tissue, but in this case the size of the observed NPs is significantly smaller than in previous cases. Further z-scan 3D reconstructions can be found as SI.

### 3.5 Up-conversion model

The green UC efficiency of Yb:Er:DMo/DW compounds is extraordinary large for what usually observed in oxides with large cutoff phonon energy. For NaT(WO_4_)_2_ (T = Y, La, Gd) ħω = 917, 923, 919 cm^-1^, respectively.[51,52] In fact, the green UC efficiency in Yb:Er:DMo/DW has been shown above to be as large as that found for the Yb:Er:β-NaYF_4_ reference compound.

**Fig 8:**
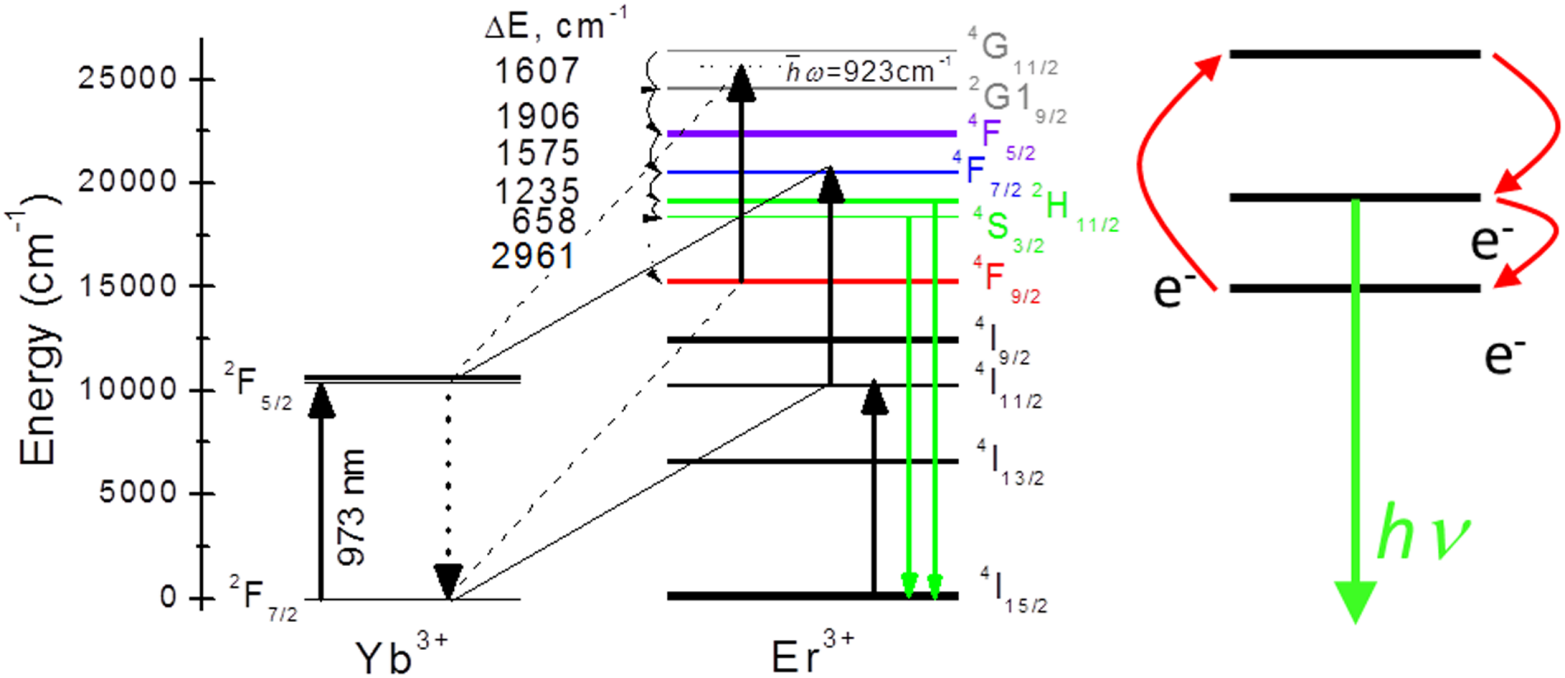
Physical model of up-conversion. Energy level diagram of the Yb-Er excitation and energy transfer mechanisms giving rise to green UC in DMo/DW compounds. Note that the phonon assisted Yb^3+^(^2^F_5/2_→^2^F_7/2_)+ħω(≈923 cm^-1^)-Er^3+^(^4^F_9/2_→^2^G_11/2_) transition bleaches the red UC emission. This electron recycling is further illustrated in the right hand inset.

Er^3+^ UC may arise from two sequential intraionic absorptions (^4^I_15/2_→^4^I_11/2_→^4^F_7/2_), however this process has low efficiency because of the small ground state absorption cross section of the ^4^I_15/2_ level (σ_ABS_≈ 0.3×10^−20^ cm^2^ for DMo/DW). Much more efficient excitation is obtained by Yb^3+^ (donor) photon absorption (in NaY(WO_4_)_2_ σ_ABS_ = 1.84×10^−20^ and 2.52×10^−20^ cm^2^ for σ and π polarizations, respectively) and subsequent resonant energy transfer to Er^3+^ (acceptor), see Fig 8. For efficient energy transfer good resonance between the donor and acceptor energy levels is needed. DMo/DW compounds fulfill this requirement with very good accuracy. The energy levels of Yb^3+^ and Er^3+^ have been reported in detail for several DMo/DW single crystals using low temperature (<10 K) spectroscopy.[46,53] Since the changes from host to host of these isostructural compounds are minor, we shall take as reference the case of NaY(WO_4_)_2_.[46]

The most intense Yb^3+^ ground state absorption in DMo/DW occurs for the 0→0´ transition with maximum at 973 nm (10277.5 cm^-1^). It is interesting to note that the energy of the Er^3+ 4^I_11/2_ multiplet extends from 10200 to 10300 cm^-1^, thus a perfect Yb^3+^(^2^F_5/2_→^2^F_7/2_)-Er^3+^(^4^I_15/2_→^4^I_11/2_) resonance occurs. Moreover, the energy gap between Er^3+ 4^I_11/2_ and ^4^F_7/2_ multiplets in DMo/DW is 10276 cm^-1^, providing again a perfect resonance for the Yb^3+^(^2^F_5/2_→^2^F_7/2_)-Er^3+^(^4^I_11/2_→^4^F_7/2_) energy transfer. Standardly, in oxides the energy gaps between consecutive Er^3+^ multiplets below ^4^F_7/2_ are less than the total energy of two or three cutoff phonons, thus all these multiplets are populated by non radiative multiphonon relaxations[54] giving rise to blue (Er^3+^, ^4^F_7/2_→^4^I_15/2_), green (Er^3+^, ^2^H_11/2_+^4^S_3/2_→^4^I_15/2_), red (Er^3+^, ^4^F_9/2_→^4^I_15/2_), and infrared (Er^3+^, ^4^I_9/2_→^4^I_15/2_) UC emissions, notably with competitive intensities between green and red UC channels.[55,56]

What is different in DMo/DW hosts is the extremely small intensity of red UC emission. This indicates that after non-radiative relaxation from ^4^S_3/2_ to ^4^F_9/2_, the almost complete depopulation of this red emitting level is achieved either by non radiative electronic relaxation or through electron recycling to upper excited states. Taking into account that the energy gap between ^4^F_9/2_ and ^4^G_11/2_ multiplets is in the 10895–11251 cm^-1^ range, it seems very likely that a phonon assisted resonant energy transfer Yb^3+^(^2^F_5/2_→^2^F_7/2_)+ħω(≈923 cm^-1^)-Er^3+^(^4^F_9/2_→^4^G_11/2_) and subsequent relaxation to ^2^H_11/2_ and ^4^S_3/2_ takes place, being this the origin of the large green UC efficiency found in Yb:Er:DMo/DW hosts. This electron recycling is illustrated in the inset of Fig 8.

**Fig 9:**
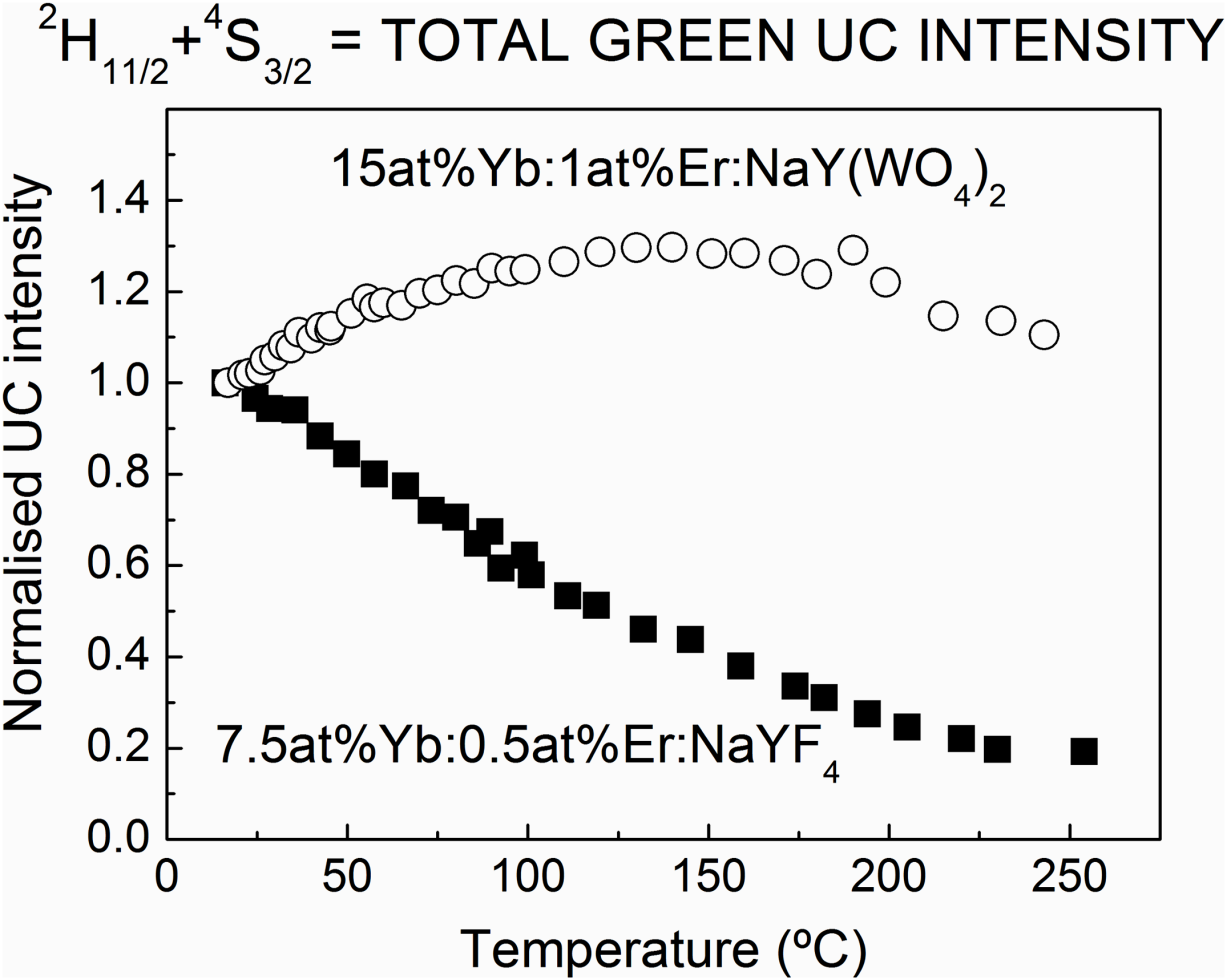
Thermal up-conversion efficiency change. Thermal evolution comparison of the integrated ^2^H_11/2_+^4^S_3/2_ green UC emission intensity from 15at%Yb:1at%Er:NaY(WO_4_)_2_ (○) and 7.5at%Yb:0.5at%Er:NaYF_4_ (■) compounds. In both cases the intensity at 300 K has been normalized to one.

This proposal is further supported by the study of the thermal evolution of the total ^2^H_11/2_+^4^S_3/2_ UC emission in Yb:Er:NaY(WO_4_)_2_ and Yb:Er:β-NaYF_4_, shown in Fig 9. While the total green UC intensity monotonously decreases with increasing temperature for Yb:Er:β-NaYF_4_, in the Yb:Er:NaY(WO_4_)_2_ case it increases from RT to 150°C and decays only for higher temperature. This shows that in the fluoride non radiative losses dominates the UC rate while in the considered oxide hosts the non radiative losses are diminished by host thermal energy transferred to excited electrons. Moreover, the n exponent of the I_UC_~I_P_^n^ relationship, which is often identified with the number of photons involved in the UC excitation mechanism is larger for NaY(WO_4_)_2_ than for β-NaYF_4_, see SI.

## 4. Conclusions

Yb:Er:NaT(XO_4_)_2_ (T = Y or Ln, and X = Mo or W) compounds with crystalline disorder over the Na and T lattice sites exhibit green UC efficiency comparable to that of Yb:Er:β-NaYF_4_, generally accepted as reference compound for UC processes. This occurs in spite of the large cutoff phonon energy of these oxides (≈ 920 cm^-1^) which is about twice of that corresponding to the fluoride. It is suggested that the principal origin of such remarkable efficiency for an oxide host is the recycling of electrons in the ^4^F_9/2_ Er^3+^ multiplet by a phonon assisted transition to the ^4^G_11/2_ one. Such high UC yield in Yb:Er:DMo/DW allowed the subcutaneous testing of *ex-vivo* chicken breast with thickness similar (in excess of 2 mm) to that achieved with Yb:Er:β-NaYF_4_ material. Individual quasi-spherical nanoparticles with diameters in the 50–80 nm range and good crystalline quality have been obtained by the sol-gel method when the calcination temperature and time is limited to 600°C and 12 h, respectively, to prevent particle clustering and sintering observed at higher temperatures (700–800°C) and times (> 12 h). These experimental conditions are more easily fulfilled by NaT(MoO_4_)_2_ compounds having a high phase purity even with 600°C calcination, while NaT(WO_4_)_2_ counterparts require larger calcination temperature and time to fully eliminate the nucleation of Na_2_W_2_O_7_ phase. The UC signal of 25at%Yb:5at%Er:NaLu(MoO_4_)_2_ sol-gel NPs perfused in a mouse has been unequivocally identified by using fluorescence lifetime imaging microscopy (FLIM) and further compared with the SHG or autofluorescence images of the surrounding tissue. These studies show that the present sol-gel NPs with sub-100 nm size reach all mouse organs, including heart, lung, kidney, liver, spleen, eye and brain while keeping ≈10% of the UC efficiency observed in bulk counterpart material and offering unless 3 times larger UC ratiometric thermal sensitivity than Yb:Er: β-NaYF_4_ currently used UC nanoprobes.

## Supporting information

S1 Fig Sol-gel method. Schematic chart of the preparation of NaLn_1-x-y_Yb_x_Er_y_(XO_4_)_2_ (X = Mo, W) nanoparticles by the sol-gel method.
(PDF)

S2 Fig DSC of the precursor powder. DSC-TG analysis of the precursor powder prepared for the synthesis of NaLu_0.5_Yb_0.5_(WO_4_)_2_ nanoparticles. Heat flow (continuous line) and mass change (dashed line). The sample was heated and cooled in air at a rate of 10 K/min, with an isotherm period of 1 h at 720°C.
(PDF)

S3 Fig Crystalline phases in calcinated sol-gel products. Room temperature pXRD of 25at%Yb:5at%Er:NaLu(XO_4_)_2_ sol-gel products obtained by calcination of the precursor black powder. (a) X = Mo. (b) X = W. X-ray reflections corresponding to the Na_2_W_2_O_7_ phase are labelled (*).
(PDF)

S4 Fig Morphology of sol-gel nanoparticles. TEM and HRTEM images of 25at%Yb:5at%Er:NaLu(MoO_4_)_2_ sol-gel synthesized nanoparticles after calcination at increasing temperatures and times. (a-c) Calcined at 600°C for 4h. Individual NPs are observed. (d-e) Calcined at 600°C for 12 h. A mixture of isolated and agglomerated NPs is observed. (f-h) Calcined at 700°C for 6h. Sintering of NPs is evident both in TEM and in HRTEM (right pictures). (i) Calcined at 800°C for 12h. Large scale sintering is observed.
(PDF)

S5 Fig Ultrasound treatment of nanoparticles. Effect of the ultrasonic treatment on the DLS-derived hydrodynamic size distributions of 25%Yb:5%Er:NaLu(MoO_4_)_2_ sol-gel products synthesized after 6 h calcination at 600°C. The white solid powder was dispersed in distilled water and submitted to ultrasonic vibration for different times. a) As received dispersion. b) 4 min ultrasonic treatment. c) 8 min ultrasonic treatment. d) 12 min ultrasonic treatment.
(PDF)

S6 Fig Ultrasound treatment of nanoparticles calcined at 600°C during different annealing times. DLS-derived hydrodynamic size distributions of 25%Yb:5%Er:NaLu(MoO_4_)_2_ sol-gel products obtained after different calcination times at 600°C and subsequently treated 12 min in the ultrasonic processor. Products obtained after a) 6 h, b) 8h and c) 12 h of calcination.
(PDF)

S7 Fig Thermal stability of 25at%Yb:5at%Er:NaY(WO_4_)_2_ compounds. LnR vs 1/T representation of the thermometric properties of a 25at%Yb:5at%Er:NaY(WO_4_)_2_ powdered sample prepared by solid state reaction. Three (stars, squares, triangles) consecutive heating (open symbols) /cooling (full symbols) cycles have been measured to show the thermal stability of the sample and to evaluate the reproducibility of the thermometric measurements. The line is the fit, LnR = 3.73–1277.85(1/T) (*C* = 41.7), of the whole data set providing *S*(317 K) = 99×10^−4^ K^-1^.
(PDF)

S8 Fig Dependence of determined up-conversion ratiometric thermal sensitivity with heating plate-sensing thermocouple union distance. LnR vs 1/T representations obtained at three distances (h = 1.25 squares, 1.9 mm triangles, 2.4 mm stars) between the heating plate and the tip of the thermocouple buried to determine the temperature of a 25at%Yb:5at%Er:NaY(WO_4_)_2_ powdered sample prepared by solid state reaction. The lines are the fits for different data sets: h = 1.25 mm, black line, LnR = 3.614–1283.56(1/T), *S*(317 K) = 82.7×10^−4^ K^-1^; h = 1.9 mm, red line, LnR = 3.47–1227.92(1/T), *S*(317 K) = 81.6×10^−4^ K^-1^; and h = 2.4 mm, blue line, LnR = 3.39–1201.68(1/T), *S*(317 K) = 80.2×10^−4^ K^-1^.
(PDF)

S9 Fig Thermometry of sol-gel nanoparticles. LnR vs 1/T representation of the thermometric properties of 25at%Yb:5at%Er:NaLu(MoO_4_)_2_ sub-100 nm nanoparticles prepared by sol-gel (calcined 12 h at 650°C and dispersed by ultrasounds). The particles were dispersed in distilled water for fluorescence measurements. The data fit provides LnR = 3.44–1020.83(1/T), C = 31.3, *S*(317K) = 127x10^-4^ K^-1^.
(PDF)

S10 Fig Evolution of up-conversion with power excitation. Evolution of the UC emission intensity (I_UC_) as a function of the pump intensity (I_P_) for 15at%Yb:1at%Er:NaY(WO_4_)_2_ (red, Δ) and 7.5at%Yb:0.5at%Er:NaYF_4_ (black, ▼) compounds at (a) T = 20°C and (b) T = 150°C. The lines are the fits used to calculate the n exponent of the I_UC_~I_P_^n^ relationship in the logarithmic representation.
(PDF)

S11 Fig Phasor FLIM analysis. Geometric bidimensional representation of the lifetime distribution of a digital image.
(PDF)

S12 Fig Bidimensional image composition. (a) Autofluorescence image of a lung section. λ_EXC_ = 850 nm, λ_EMI_ = 600–650 nm. (b) UC image of NPs distributed in the same tissue area acquired λ_EXC_ = 973 nm, λ_EMI_ = 535 nm. (c) Composite image that allows the localization of the particles. Images are represented in pseudo-color normalized intensity scales: NPs in green and tissues in red.
(PDF)

S13 Fig Three dimensional image composition. 3D rendering of z-scans showing the penetration of UC (pseudo-color green scale) inside a fragment of kidney, brain and liver tissues (pseudo-color red autofluorescence scale) after perfusion of the mouse with a PBS dispersion of sol-gel synthesized 25at%Yb:5at%Er:NaLu(MoO_4_)_2_ NPs (calcined at 600°C for 12 h).
(PDF)
