# Supplementary material for: Efficient up-conversion in Yb:Er:NaT(XO4)2 thermal nanoprobes. Imaging of their distribution in a perfused mouse
Source: PLoS One. 2017 May 18;12(5):e0177596. doi: 10.1371/journal.pone.0177596 (PMC5436681; doi:10.1371/journal.pone.0177596)
Supplement: S1 Fig — Schematic chart of the preparation of NaLn1-x-yYbxEry(XO4)2 (X = Mo, W) nanoparticles by the sol-gel method. (PDF) [file pone.0177596.s001.pdf]

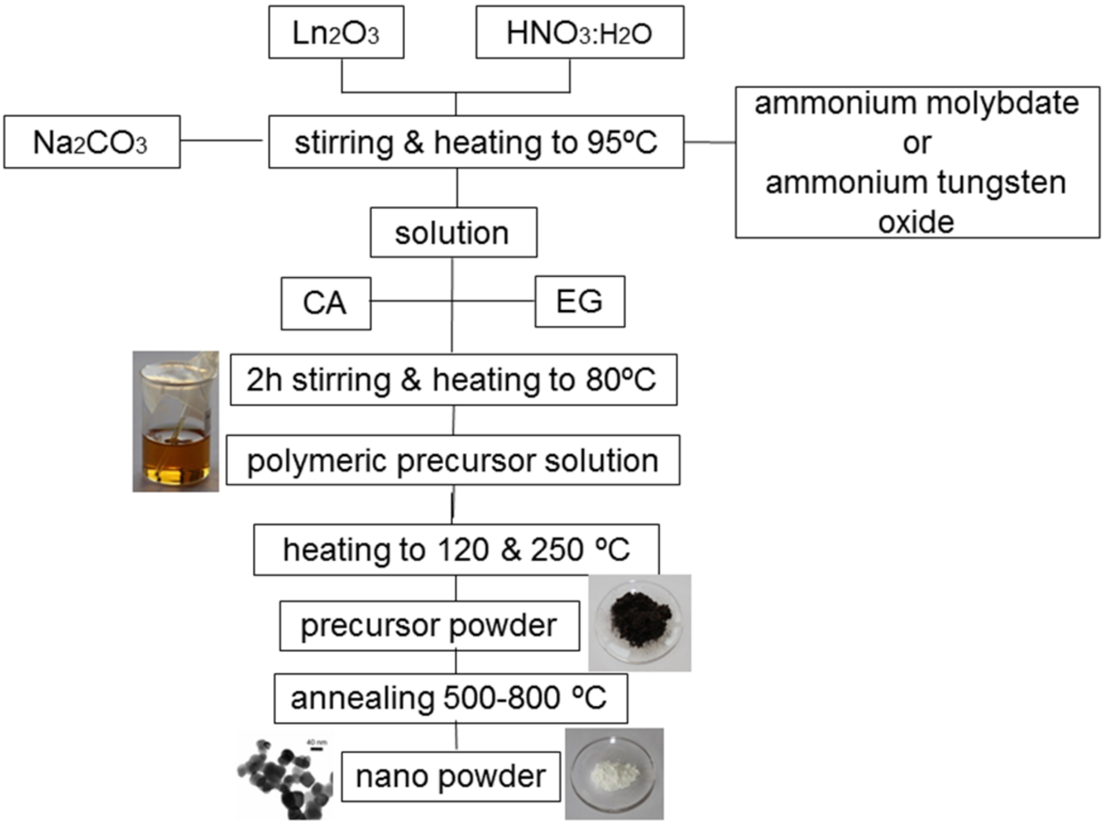

S1 Fig. Sol-gel method. Schematic chart of the preparation of $NaLn_{1-x-y}Yb_xEr_y(XO_4)_2$ (X= Mo, W) nanoparticles by the sol-gel method.

S1 Fig sketches the sol-gel procedures used for nanoparticle (NP) synthesis. The precursor chemicals used were Na_2CO_3 (99.5%), Y_2O_3, Ln_2O_3, (Ln= La, Gd, Er, Yb, Lu, 99.99%), $(NH_4)_6Mo_7O_{24}\cdot 4H_2O$, $(NH_4)_6H_2W_{12}O_{40}\cdot nH_2O$, HNO_3 (69 wt%), citric acid (HOOC-CH_2-(OH)(COOH)-CH_2-COOH, CA) and ethylene glycol ($HOCH_2$-CH_2OH, EG). Proper amounts of raw Y_2O_3 or Ln_2O_3 were dissolved in diluted HNO_3 and a suitable volume of deionized water under vigorous stirring and heating at around 90 ºC, after the solution was clear, heating continued under stirring to evaporate the extra HNO_3. Afterwards, required amounts of Na_2CO_3 and ammonium molybdenum (or tungsten) oxide hydrate were added to the solution. Then, citric acid (hereafter CA) was added with some extra water until full dissolution. Further, EG was added as complexing agent. The above mixture was stirred for 2 hours more at about 80 ºC to get a stable and optimized precursor. Lastly, ammonia（30 wt%）was added drop by drop to adjust the pH value to the 4-5 range and the solution was stirred for one hour more.

The main disadvantage of the sol-gel Pechini method for preparation of inorganic NPs is that the resin used for synthesis contains a large amount of organic materials which incorporates during the polymerization process. Thus an annealing under oxygen rich atmosphere must be applied to remove the organic constituents leaving only the metals of the desired ceramic composition. This is made in two steps: i) The polymeric precursor solution is dried first to 120 ºC and later to 250 ºC obtaining a black powder precursor; ii) this powder is subsequently calcined to higher temperature.
