# Supplementary material for: Efficient up-conversion in Yb:Er:NaT(XO4)2 thermal nanoprobes. Imaging of their distribution in a perfused mouse
Source: PLoS One. 2017 May 18;12(5):e0177596. doi: 10.1371/journal.pone.0177596 (PMC5436681; doi:10.1371/journal.pone.0177596)
Supplement: S2 Fig — DSC-TG analysis of the precursor powder prepared for the synthesis of NaLu0.5Yb0.5(WO4)2 nanoparticles. Heat flow (continuous line) and mass change (dashed line). The sample was heated and cooled in air at a rate of 10 K/min, with an isotherm period of 1 h at 720°C. (PDF) [file pone.0177596.s002.pdf]

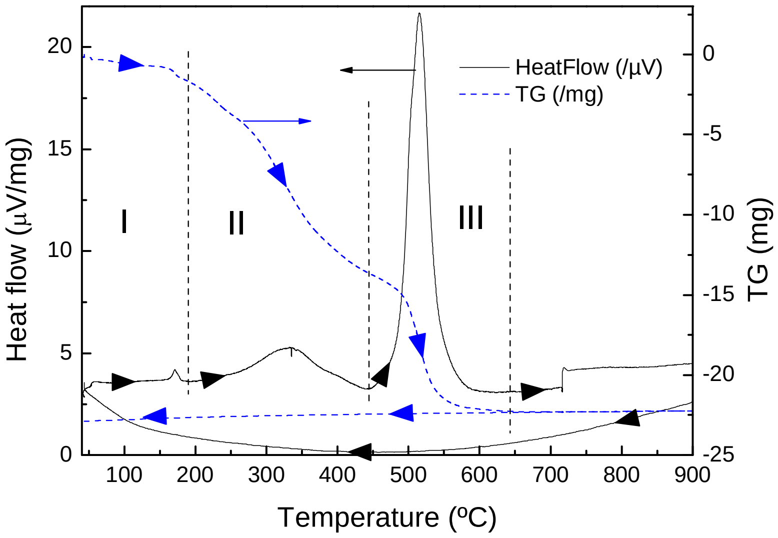

S2 Fig. DSC of the precursor powder. DSC-TG analysis of the precursor powder prepared for the synthesis of $NaLu_{0.5}Yb_{0.5}(WO_4)_2$ nanoparticles. Heat flow (continuous line) and mass change (dashed line). The sample was heated and cooled in air at a rate of 10 K/min, with an isotherm period of 1 h at 720 ºC.

S2 Fig shows the DSC-TG thermogram of a $NaLu_{0.5}Yb_{0.5}(WO_4)_2$ precursor powder prepared by the above described method. The thermal decomposition of the precursor solution can be divided into three stages associated with the weight loss. Stage I, 40-190 ºC including a weak exothermic peak at 171 ºC, and stage II, 190-445 ºC including a broad exothermic peak with maximum at 332 ºC, can be attributed to the oxidation of residual CA and EG. The combustion of residual nitrate may also contribute to the peak at 332 ºC. Stage III, 445-640 ºC including the strong exothermic peak with maximum at 515 ºC, corresponds to the decomposition/combustion of the polyester and also to the crystallization process because the latter involves an exothermic transformation without mass loss. The overall weight loss was of 76.9 % of the initial weight. Heating above 650 ºC does not induce further weight loss. These combustion processes impose the 500-550 ºC temperature limit as the lowest calcination temperature for the synthesis of nanoparticles.
