# Supplementary material for: Efficient up-conversion in Yb:Er:NaT(XO4)2 thermal nanoprobes. Imaging of their distribution in a perfused mouse
Source: PLoS One. 2017 May 18;12(5):e0177596. doi: 10.1371/journal.pone.0177596 (PMC5436681; doi:10.1371/journal.pone.0177596)
Supplement: S3 Fig — Room temperature pXRD of 25at%Yb:5at%Er:NaLu(XO4)2 sol-gel products obtained by calcination of the precursor black powder. (a) X = Mo. (b) X = W. X-ray reflections corresponding to the Na2W2O7 phase are labelled (*). (PDF) [file pone.0177596.s003.pdf]

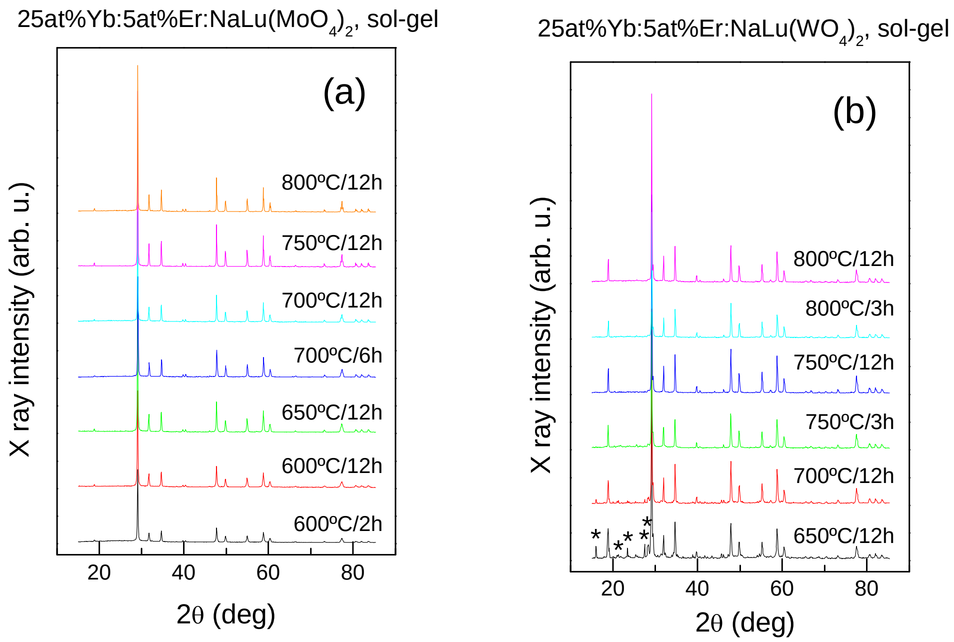

S3 Fig. Crystalline phases in calcinated sol-gel products. Room temperature pXRD of 25at%Yb:5at%Er:NaLu(XO_4)$_2$ sol-gel products obtained by calcination of the precursor black powder. (a) X= Mo. (b) X= W. X-ray reflections corresponding to the $Na_2W_2O_7$ phase are labelled (*).

The nature of the products obtained after calcination depends on calcination temperature and time as well as on the Mo or W composition of the pursuit material. S3 Fig shows room temperature (RT) powder X-ray diffraction (pXRD) studies performed after calcination. NaT(MoO_4)$_2$ compounds appear free of second phases independently of the calcination temperature and time. Only the tetragonal scheelite phase is observed. As an example S3a Fig shows these results for 25at%Yb:5at%Er:NaLu(MoO_4)$_2$. In contrast to this situation the sol-gel products of NaT(WO_4)$_2$ intended compounds show at the lowest calcination temperature and time the presence of a second phase which has been identified as $Na_2W_2O_7$. Most intense pXRD reflections corresponding to this undesired phase are labelled in S3b Fig.
