# Supplementary material for: Efficient up-conversion in Yb:Er:NaT(XO4)2 thermal nanoprobes. Imaging of their distribution in a perfused mouse
Source: PLoS One. 2017 May 18;12(5):e0177596. doi: 10.1371/journal.pone.0177596 (PMC5436681; doi:10.1371/journal.pone.0177596)
Supplement: S4 Fig — TEM and HRTEM images of 25at%Yb:5at%Er:NaLu(MoO4)2 sol-gel synthesized nanoparticles after calcination at increasing temperatures and times. (a-c) Calcined at 600°C for 4h. Individual NPs are observed. (d-e) Calcined at 600°C for 12 h. A mixture of isolated and agglomerated NPs is observed. (f-h) Calcined at 700°C for 6h. Sintering of NPs is evident both in TEM and in HRTEM (right pictures). (i) Calcined at 800°C for 12h. Large scale sintering is observed. (PDF) [file pone.0177596.s004.pdf]

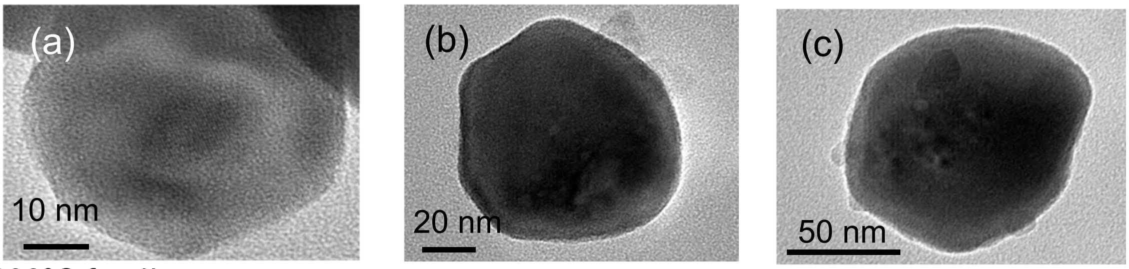

600ºC for 4h.

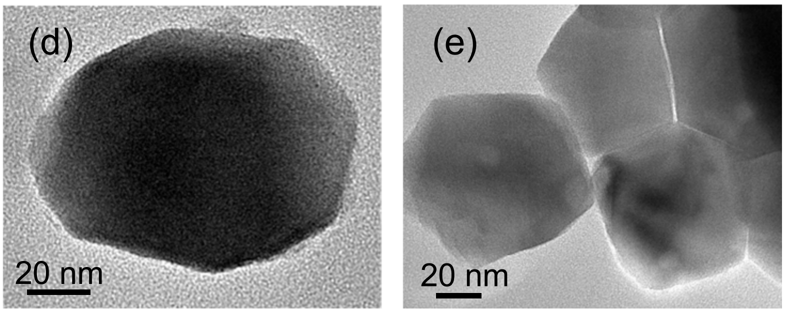

600ºC for 12h.

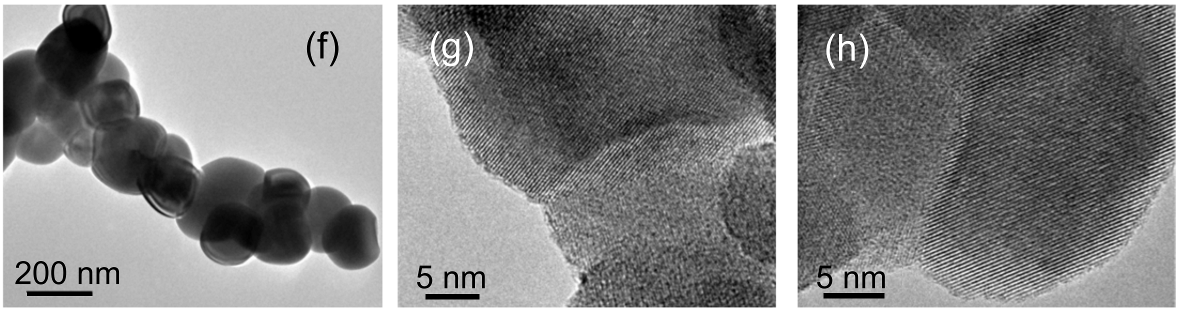

700ºC 6h

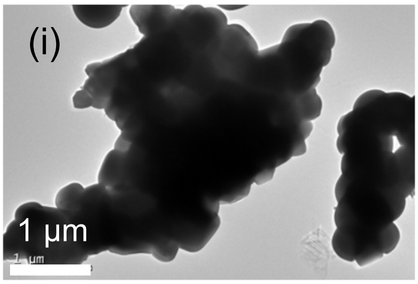

800ºC for 12h.

S4 Fig. Morphology of sol-gel nanoparticles. TEM and HRTEM images of 25at%Yb:5at%Er:NaLu$(MoO_4)_2$ sol-gel synthesized nanoparticles after calcination at increasing temperatures and times. (a-c) Calcined at 600 ºC for 4h. Individual NPs are observed. (d-e) Calcined at 600 ºC for 12 h. A mixture of isolated and agglomerated NPs is observed. (f-h) Calcined at 700 ºC for 6h. Sintering of NPs is evident both in TEM and in HRTEM (right pictures). (i) Calcined at 800 ºC for 12h. Large scale sintering is observed.

The calcination temperature and time have further effect on the resulting products. S4 Fig shows a transmission electron microscopy (TEM) study, including high resolution (HRTEM) images, of the 25at%Yb:5at%Er:NaLu$(MoO_4)_2$ compound. For the lowest temperature and time monitored (600 ºC, 4 h) individual particles with sizes in the 50-80 nm range are easily observed, see S4a, S4b and S4c Figs. The increase of the calcination time even at this relatively low calcination temperature promotes some incipient particle sintering, see S4d and S4e Figs, but grain boundaries are yet not clearly observed in HRTEM. Higher calcination temperature (700 ºC) clearly shows particle sintering forming rows, see S4f Fig, and HRTEM shows evidence of the development of grain boundaries, see S4g and S4h Figs. Particle sintering grows with temperature. At the largest calcination temperature and time studied (800 ºC, 12 h) large scale particle aggregation and sintering is observed, see S4h Fig, forming clusters larger than 1 μm.
