# Supplementary material for: Efficient up-conversion in Yb:Er:NaT(XO4)2 thermal nanoprobes. Imaging of their distribution in a perfused mouse
Source: PLoS One. 2017 May 18;12(5):e0177596. doi: 10.1371/journal.pone.0177596 (PMC5436681; doi:10.1371/journal.pone.0177596)
Supplement: S5 Fig — Effect of the ultrasonic treatment on the DLS-derived hydrodynamic size distributions of 25%Yb:5%Er:NaLu(MoO4)2 sol-gel products synthesized after 6 h calcination at 600°C. The white solid powder was dispersed in distilled water and submitted to ultrasonic vibration for different times. a) As received dispersion. b) 4 min ultrasonic treatment. c) 8 min ultrasonic treatment. d) 12 min ultrasonic treatment. (PDF) [file pone.0177596.s005.pdf]

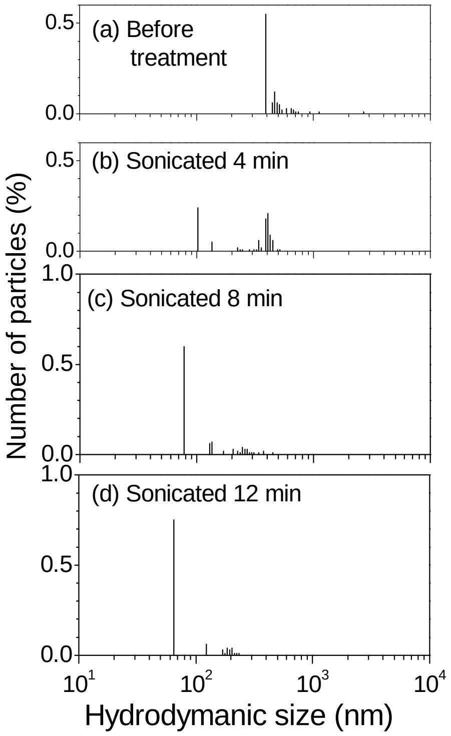

S5 Fig. Ultrasound treatment of nanoparticles. Effect of the ultrasonic treatment on the DLS-derived hydrodynamic size distributions of 25%Yb:5%Er:NaLu$(MoO_4)_2$ sol-gel products synthesized after 6 h calcination at 600 ºC. The white solid powder was dispersed in distilled water and submitted to ultrasonic vibration for different times. a) As received dispersion. b) 4 min ultrasonic treatment. c) 8 min ultrasonic treatment. d) 12 min ultrasonic treatment.

The hydrodynamic size distribution of the sol-gel products dispersed in water has been determined by dynamic light scattering (DLS) technique. S5 Fig shows the size distribution of 25%Yb:5%Er:NaLu$(MoO_4)_2$ sol-gel products obtained by 6 h calcination at 600 ºC. The products obtained just after calcination contain particle clusters, 55% of them have a minimum diameter of 390 nm and the rest has larger sizes most of them up to 750 nm, and even 3% of the clusters have a size around 1 µm, see S5a Fig. These clusters are disaggregated by treatment of water particle dispersions with an ultrasonic processor, see S5b-S5d Figs. Sonication during 4 min removes all clusters with size larger than 510 nm, and induces the presence of a 25% of NPs with size in the 100-130 nm range. Dispersions treated with the ultrasonic processor for 8 min contain a 60% of NPs with 77 nm and the maximum cluster size observed is 447 nm. Finally, after 12 min of ultrasonic treatment 75% of the NPs have a hydrodynamic size of 65 nm and the maximum cluster size observed is reduced to 235 nm. These results

unequivocally show that the 25%Yb:5%Er:$NaLu(MoO_4)_2$ NPs in the clusters obtained after calcination at 600 ºC are not sintered and can be dispersed by ultrasonic treatment. This conclusion agrees the previously shown TEM results. The smallest hydrodymanic sizes observed, 65-80 nm, agree the size of the individual NPs observed by HRTEM, 50-80 nm, and are also consistent with the crystalline domain size observed for these preparation conditions, ≈40 nm.
