# Supplementary material for: Efficient up-conversion in Yb:Er:NaT(XO4)2 thermal nanoprobes. Imaging of their distribution in a perfused mouse
Source: PLoS One. 2017 May 18;12(5):e0177596. doi: 10.1371/journal.pone.0177596 (PMC5436681; doi:10.1371/journal.pone.0177596)
Supplement: S6 Fig — DLS-derived hydrodynamic size distributions of 25%Yb:5%Er:NaLu(MoO4)2 sol-gel products obtained after different calcination times at 600°C and subsequently treated 12 min in the ultrasonic processor. Products obtained after a) 6 h, b) 8h and c) 12 h of calcination. (PDF) [file pone.0177596.s006.pdf]

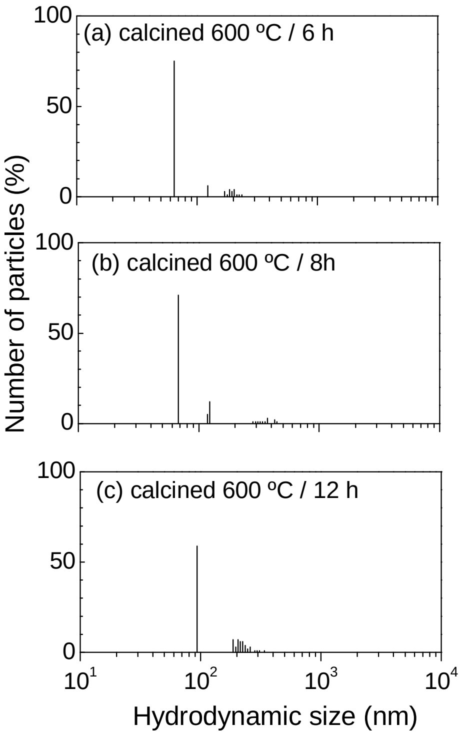

S6 Fig. Ultrasound treatment of nanoparticles calcined at 600ºC during different annealing times. DLS-derived hydrodynamic size distributions of 25%Yb:5%Er:NaLu(MoO_4)$_2$ sol-gel products obtained after different calcination times at 600 ºC and subsequently treated 12 min in the ultrasonic processor. Products obtained after a) 6 h, b) 8h and c) 12 h of calcination.

Although cluster disaggregation is observed for all used calcination times at 600 ºC, its kinetic slows as the calcination time increases. S6 Fig shows this for dispersions of particles obtained after different calcination times and subjected to the same time (12 min) of ultrasonic treatment. While the smallest particles (of 65 nm) observed in the products calcined by 6 h supposed 75% of the total observed particles, the products calcined during 12 h only achieved 59% of particles with a minimum size of 93 nm for the same time of ultrasonic treatment. Thus the sonication time for cluster disaggregation must be increased as the time for calcination grows.
