# Supplementary material for: Efficient up-conversion in Yb:Er:NaT(XO4)2 thermal nanoprobes. Imaging of their distribution in a perfused mouse
Source: PLoS One. 2017 May 18;12(5):e0177596. doi: 10.1371/journal.pone.0177596 (PMC5436681; doi:10.1371/journal.pone.0177596)
Supplement: S7 Fig — LnR vs 1/T representation of the thermometric properties of a 25at%Yb:5at%Er:NaY(WO4)2 powdered sample prepared by solid state reaction. Three (stars, squares, triangles) consecutive heating (open symbols) /cooling (full symbols) cycles have been measured to show the thermal stability of the sample and to evaluate the reproducibility of the thermometric measurements. The line is the fit, LnR = 3.73–1277.85(1/T) (C = 41.7), of the whole data set providing S(317 K) = 99×10−4 K-1. (PDF) [file pone.0177596.s007.pdf]

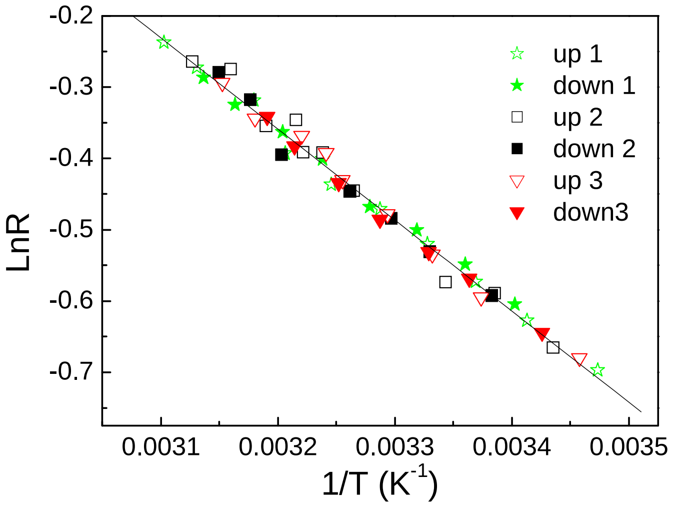

S7 Fig. Thermal stability of 25at%Yb:5at%Er:NaY(WO$_4$)$_2$ compounds. LnR vs 1/T representation of the thermometric properties of a 25at%Yb:5at%Er:NaY(WO$_4$)$_2$ powdered sample prepared by solid state reaction. Three (stars, squares, triangles) consecutive heating (open symbols) /cooling (full symbols) cycles have been measured to show the thermal stability of the sample and to evaluate the reproducibility of the thermometric measurements. The line is the fit, LnR= 3.73-1277.85(1/T) (*C*= 41.7), of the whole data set providing *S*(317 K)= 99×10^{-4} K^{-1}

For a given temperature and material, the UC ratiometric thermal sensitivity, *S*, is exclusively related to the energy gap between the Er^{3+} $^4S_{3/2}$ and $^2H_{11/2}$ multiplets, thus it represents a material property. However, when producing a thermal sensor device an important parameter to determine is the thermal resolution, *r*, which represents the minimum temperature change that the device can determine. The resolution is defined as the ratio of the uncertainty in the R measurement, σ, and the sensitivity, i.e. $r= \sigma_R/S$. Obviously *r* depends on the optical detection conditions and on the accuracy of material temperature determination during calibration, thus the comparison between *r* values of different works is not straightforward.

In order to show the limits and reproducibility of the UC ratiometric thermometry results presented in the main text, we have made further thermometric characterizations. S7 Fig shows the characteristic LnR versus 1/T plot for three consecutive heating/cooling cycles of a 25at%Yb:5at%Er:NaY(WO$_4$)$_2$ powdered sample synthesized by solid state reaction. The results depend very little on the heating or cooling regimes and are close each other for the three cycles. The UC ratiometric thermal sensitivity and resolution determined at 317 K (44ºC) from the whole (six runs) data set are *S*= 99×10^{-4} K^{-1} and *r*= 0.9 K, respectively. These measurements show the thermal stability of the used DW.
