# Supplementary material for: Efficient up-conversion in Yb:Er:NaT(XO4)2 thermal nanoprobes. Imaging of their distribution in a perfused mouse
Source: PLoS One. 2017 May 18;12(5):e0177596. doi: 10.1371/journal.pone.0177596 (PMC5436681; doi:10.1371/journal.pone.0177596)
Supplement: S8 Fig — LnR vs 1/T representations obtained at three distances (h = 1.25 squares, 1.9 mm triangles, 2.4 mm stars) between the heating plate and the tip of the thermocouple buried to determine the temperature of a 25at%Yb:5at%Er:NaY(WO4)2 powdered sample prepared by solid state reaction. The lines are the fits for different data sets: h = 1.25 mm, black line, LnR = 3.614–1283.56(1/T), S(317 K) = 82.7×10−4 K-1; h = 1.9 mm, red line, LnR = 3.47–1227.92(1/T), S(317 K) = 81.6×10−4 K-1; and h = 2.4 mm, blue line, LnR = 3.39–1201.68(1/T), S(317 K) = 80.2×10−4 K-1. (PDF) [file pone.0177596.s008.pdf]

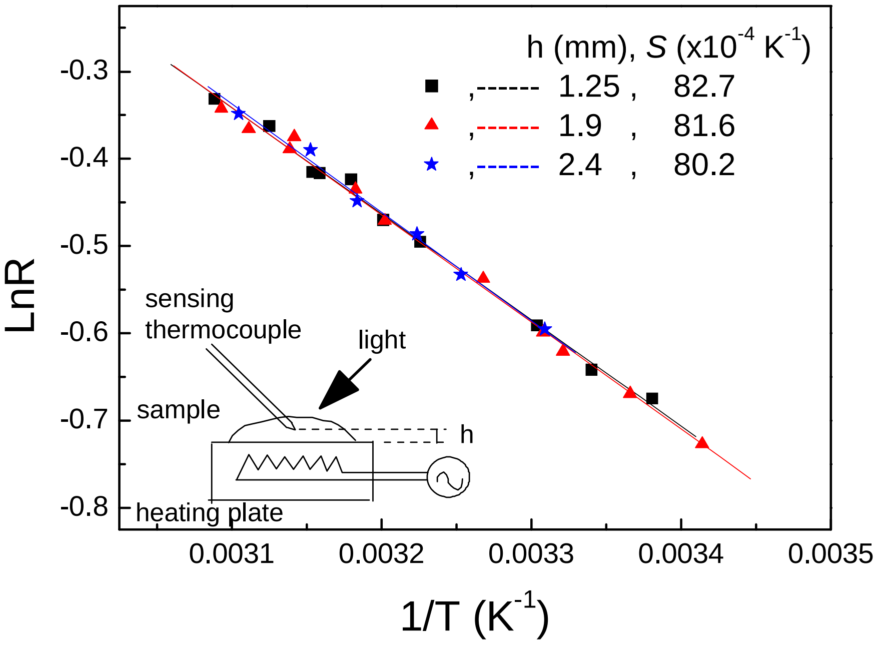

S8 Fig. Dependence of determined upconversion ratiometric thermal sensitivity with heating plate-sensing thermocouple union distance. LnR vs 1/T representations obtained at three distances (h= 1.25 squares, 1.9 mm triangles, 2.4 mm stars) between the heating plate and the tip of the thermocouple buried to determine the temperature of a 25at%Yb:5at%Er:NaY$(WO_4)_2$ powdered sample prepared by solid state reaction. The lines are the fits for different data sets: h=1.25 mm, black line, LnR=3.614-1283.56(1/T), *S*(317 K)= 82.7×10^{-4} K^{-1}; h=1.9 mm, red line, LnR=3.47-1227.92(1/T), *S*(317 K)= 81.6×10^{-4} K^{-1}; and h=2.4 mm, blue line, LnR=3.39-1201.68(1/T), *S*(317 K)= 80.2×10^{-4} K^{-1}.

Another source of uncertainty in the evaluation of the ratiometric thermal sensitivity is the measurement of the real temperature of the tested sample due to low thermal conductivity of the insulating compounds. In order to realize the influence of heat diffusion in MT$(XO_4)_2$ powdered samples, instead to trust in the nominal temperature of the resistive heating plate we buried an independent sensing thermocouple in the tested powder and controlled the vertical distance between the tip of the thermocouple and the surface of the heating plate. S8 Fig shows the *S* differences encountered for three tested distances. No large differences are found, but still a systematic reduction of the determined UC ratiometric thermal sensitivity is observed with increasing heating/sensing distance. These results show that trusting in the plate nominal temperature overestimates *S*, what is surprising since the powder surface from

which the UC is collected is expected to be at slightly lower temperature than the heating plate. One possible reason for this finding is that despite the precautions taken (low irradiation power density and pulsed regime) the sample surface was overheated by the pumping laser.
