# Supplementary material for: Efficient up-conversion in Yb:Er:NaT(XO4)2 thermal nanoprobes. Imaging of their distribution in a perfused mouse
Source: PLoS One. 2017 May 18;12(5):e0177596. doi: 10.1371/journal.pone.0177596 (PMC5436681; doi:10.1371/journal.pone.0177596)
Supplement: S9 Fig — LnR vs 1/T representation of the thermometric properties of 25at%Yb:5at%Er:NaLu(MoO4)2 sub-100 nm nanoparticles prepared by sol-gel (calcined 12 h at 650°C and dispersed by ultrasounds). The particles were dispersed in distilled water for fluorescence measurements. The data fit provides LnR = 3.44–1020.83(1/T), C = 31.3, S(317K) = 127x10-4 K-1. (PDF) [file pone.0177596.s009.pdf]

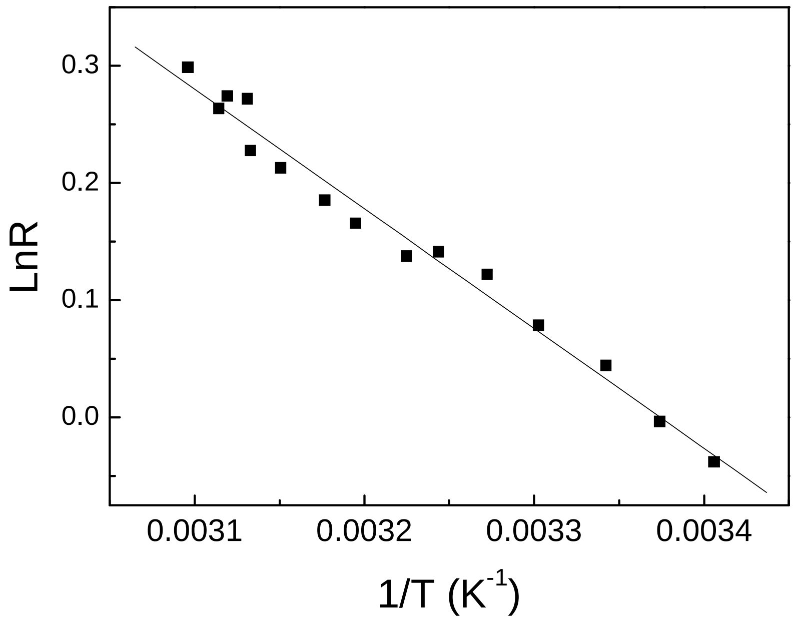

S9 Fig. Thermometry of sol-gel nanoparticles. LnR vs 1/T representation of the thermometric properties of 25at%Yb:5at%Er:$NaLu(MoO_4)_2$ sub-100 nm nanoparticles prepared by sol-gel (calcined 12 h at 650 ºC and dispersed by ultrasounds). The particles were dispersed in distilled water for fluorescence measurements. The data fit provides LnR=3.44-1020.83(1/T), C= 31.3, and *S*(317K)= $127x10^{-4}$ K^{-1}.

The ratiometric thermal sensitivity of the sol-gel synthesized 25at%Yb:5at%Er:$NaLu(MoO_4)_2$ sub-100 nm NPs perfused into the mouse has been determined for comparison with counterpart micrometric sized DMo/DW compounds. S9 Fig shows the LnR vs 1/T representation of the data. In that case the products were dispersed in distilled water for fluorescence measurements and the temperature was controlled with a recirculating water bath. *S*(317K)= $127x10^{-4}$ K^{-1} has been determined. This value seems to be significantly larger than those obtained in this work for powdered micrometric sized Yb:Er:$NaY(WO_4)_2$, and is in fact closer to those previously published for $AgLa(MoO_4)_2$, or $NaGd(MoO_4)_2$ compounds. We are skeptical that this represents a change in material properties, instead the influence of the measuring experimental conditions must be searched in more detail with particular emphasis in determining the real temperature of the tested sample upon simultaneous heating and light irradiation.
