# Supplementary material for: Efficient up-conversion in Yb:Er:NaT(XO4)2 thermal nanoprobes. Imaging of their distribution in a perfused mouse
Source: PLoS One. 2017 May 18;12(5):e0177596. doi: 10.1371/journal.pone.0177596 (PMC5436681; doi:10.1371/journal.pone.0177596)
Supplement: S10 Fig — Evolution of the UC emission intensity (IUC) as a function of the pump intensity (IP) for 15at%Yb:1at%Er:NaY(WO4)2 (red, Δ) and 7.5at%Yb:0.5at%Er:NaYF4 (black, ▼) compounds at (a) T = 20°C and (b) T = 150°C. The lines are the fits used to calculate the n exponent of the IUC~IPn relationship in the logarithmic representation. (PDF) [file pone.0177596.s010.pdf]

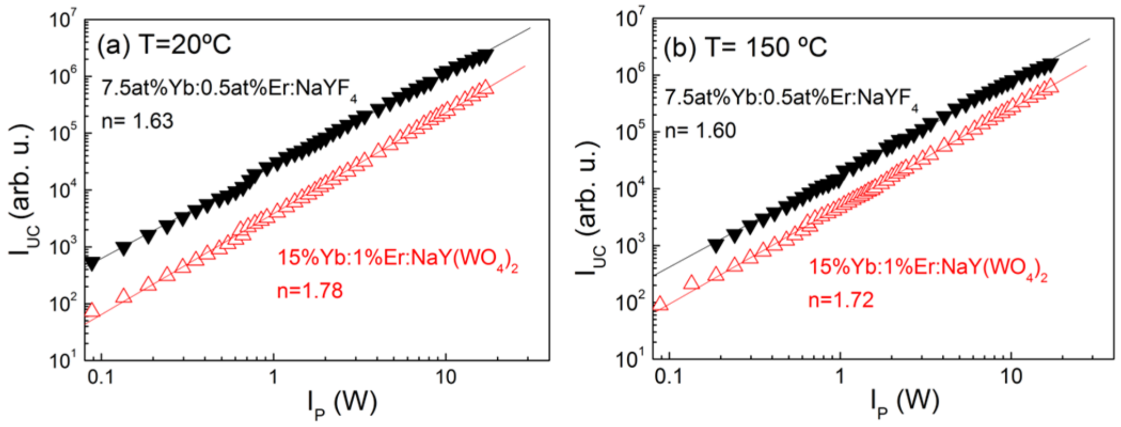

S10 Fig. Evolution of upconversion with power excitation. Evolution of the UC emission intensity (I_{UC}) as a function of the pump intensity (I_P) for 15at%Yb:1at%Er:NaY$(WO_4)_2$ (red, Δ) and 7.5at%Yb:0.5at%Er:NaYF_4 (black, ▼) compounds at (a) T= 20 ºC and (b) T= 150 ºC. The lines are the fits used to calculate the n exponent of the I_{UC}~I_P^n relationship in the logarithmic representation.

The proposed third UC excitation step, $^4F_{9/2}\rightarrow{}^4G_{11/2}$, in DMo/DW compounds arising from Yb-Er energy transfer and assisted by the absorption of a lattice phonons implies a three photon absorption mechanism for the UC. The evolution of the UC intensity (I_{UC}) with the pump intensity (I_P) is related by I_{UC}~ I_P^n, where n is in a first approximation the number of absorbed photons. However, it is well known that this situation is only reached for infinitely small UC rates. For systems producing detectable UC signal the UC intensity-versus-power dependence is always lower than I_P^n.[1] S10 Fig shows the I_{UC} vs I_P logarithmic relationships at 20 and 150 ºC for 15at%Yb:1at%Er:NaY$(WO_4)_2$ and 7.5at%Yb:0.5at%Er:NaYF_4 compounds. In all cases n<2 is found and it is observed that the exponents for Yb:Er:NaY$(WO_4)_2$ are always larger than those found for Yb:Er:NaYF_4. Although this can not be taken as a direct evidence of a three photon absorption in the UC excitation of the present oxides, it is a qualitative indication that the number of absorbed photons in the oxide case is larger than in the fluoride.

[1] Pollnau M, Gamelin DR, Luthi SR, Güdel HU, Hehlen MP (2000) Power dependence of upconversion luminescence in lanthanide and transition-metal-ion systems. Phys. Rev. B 61:3337-3346.
