# Supplementary material for: Efficient up-conversion in Yb:Er:NaT(XO4)2 thermal nanoprobes. Imaging of their distribution in a perfused mouse
Source: PLoS One. 2017 May 18;12(5):e0177596. doi: 10.1371/journal.pone.0177596 (PMC5436681; doi:10.1371/journal.pone.0177596)
Supplement: S11 Fig — Geometric bidimensional representation of the lifetime distribution of a digital image. (PDF) [file pone.0177596.s011.pdf]

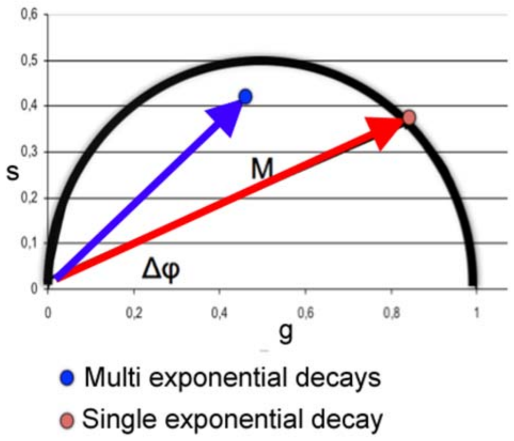

S11 Fig. Phasor FLIM analysis. Geometric bidimensional representation of the lifetime distribution of a digital image.

FLIM was performed according to the phasor FLIM method. [1-3] The fluorescence lifetime distribution was obtained by converting the time-domain multi-exponential fluorescence decays acquired in each pixel into the corresponding frequency domain response, the phase and modulation components of which are graphically represented by a phasor in polar coordinates:

$$g_i(\omega)= M_i \cos(\Delta\varphi_i) \text{ , } s_i(\omega)= M_i \sin(\Delta\varphi_i).$$

Therefore, in the phasor representation, the fluorescence decay in each pixel of the image gives a point in the phasor plot inside the so-called "universal circle", see S11 Fig. Only single exponential decays fall on the universal circle. If the decay at one pixel is a convolution of lifetimes (i.e. $\tau_{phase} \neq \tau_{modulation}$), as a result of multiple species contributing to the fluorescence intensity in that pixel, the phasor falls inside the universal circle, and it is simply the algebraic sum of phasors from each component. As previously shown, [4] the phasor transfom allows the unbiased visualization of the entire distribution of lifetime decays in a sample, without introducing any approximation on the number of exponential components of a decay in a pixel. There is a direct correspondence between the phasor plot and the FLIM image, which simply represent the localization of each convoluted lifetime value in the sample.

[1] Gratton E, Jameson DM, Hall RD (1984) Multifrequency phase and modulationfluorometry. Ann. Rev. Biophys. Bioeng. 13:105-24.

[2] Clayton AHA, Hanley QS, Verveer PJ (2004) Graphical representation and multicomponent analysis of single-frequency fluorescence lifetime imaging microscopy data. J. Microscopy 213:1-5.
[3] Redford GI, Clegg RM (2005) Polar plot representation for frequency-domain analysis of fluorescence lifetimes. J. Fluoresc. 15:805–815.
[4] Digman MA, Caiolfa V, Zamai M, Gratton E (2008) The phasor approach to fluorescence lifetime imaging analysis. Biophys. J. 94:2320–2332.
