# Supplementary material for: Efficient up-conversion in Yb:Er:NaT(XO4)2 thermal nanoprobes. Imaging of their distribution in a perfused mouse
Source: PLoS One. 2017 May 18;12(5):e0177596. doi: 10.1371/journal.pone.0177596 (PMC5436681; doi:10.1371/journal.pone.0177596)
Supplement: S12 Fig — (a) Autofluorescence image of a lung section. λEXC = 850 nm, λEMI = 600–650 nm. (b) UC image of NPs distributed in the same tissue area acquired λEXC = 973 nm, λEMI = 535 nm. (c) Composite image that allows the localization of the particles. Images are represented in pseudo-color normalized intensity scales: NPs in green and tissues in red. (PDF) [file pone.0177596.s012.pdf]

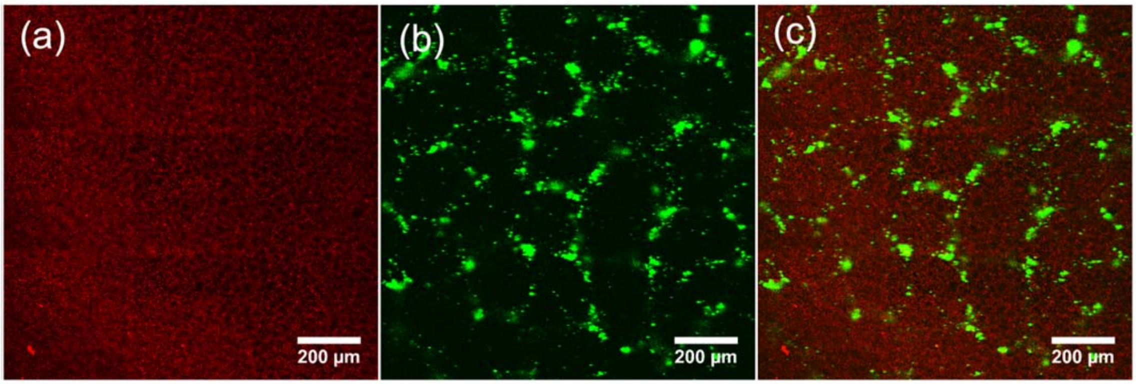

S12 Fig. Bidimensional image composition. (a) Autofluorescence image of a lung section. λ_{EXC}= 850 nm, λ_{EMI}= 600-650 nm. (b) UC image of NPs distributed in the same tissue area acquired λ_{EXC}= 973 nm, λ_{EMI}= 535 nm. (c) Composite image that allows the localization of the particles. Images are represented in pseudo-color normalized intensity scales: NPs in green and tissues in red.

Images documenting NPs distribution in mouse organs as presented in main text (Fig 7) were obtained by composing tissue autofluorescence images (S12a Fig) and UC images (S12b Fig) that were collected by sequential scans of the same tissue area. The tissue structure was best captured by autofluorescence under multiphoton excitation at λ_{EXC}= 850 nm and maximum emission at λ_{EMI}= 600-650 nm. UC were excited by single photon absorption at λ_{EXC}= 973 nm and the emission maximum at λ_{EMI}= 535 nm. S12a and S12b Figs illustrate the individual fluorescence intensity and UC images and the composite result (S12c Fig).
