# Supplementary material for: Efficient up-conversion in Yb:Er:NaT(XO4)2 thermal nanoprobes. Imaging of their distribution in a perfused mouse
Source: PLoS One. 2017 May 18;12(5):e0177596. doi: 10.1371/journal.pone.0177596 (PMC5436681; doi:10.1371/journal.pone.0177596)
Supplement: S13 Fig — 3D rendering of z-scans showing the penetration of UC (pseudo-color green scale) inside a fragment of kidney, brain and liver tissues (pseudo-color red autofluorescence scale) after perfusion of the mouse with a PBS dispersion of sol-gel synthesized 25at%Yb:5at%Er:NaLu(MoO4)2 NPs (calcined at 600°C for 12 h). (PDF) [file pone.0177596.s013.pdf]

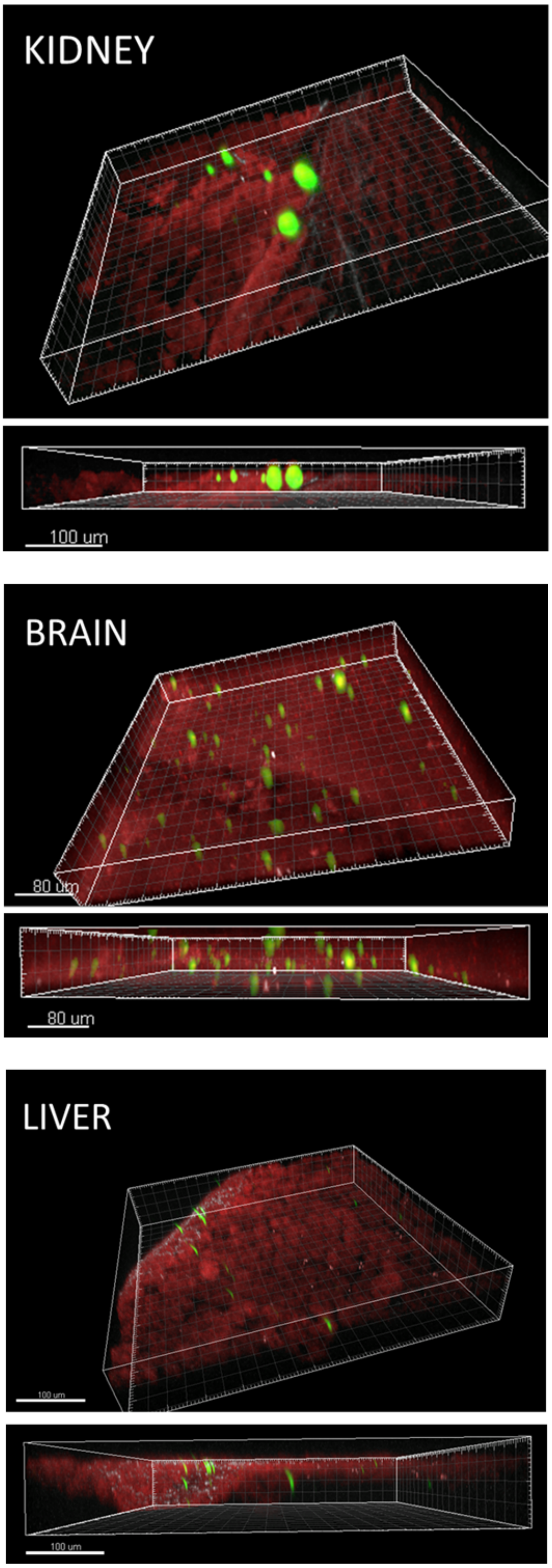

S13 Fig. Three dimensional image composition. 3D rendering of z-scans showing the penetration of UC (pseudo-color green scale) inside a fragment of kidney, brain and liver tissues (pseudo-color red autofluorescence scale) after perfusion of the mouse with a PBS dispersion of sol-gel synthesized 25at%Yb:5at%Er:$NaLu(MoO_4)_2$ NPs (calcined at 600 ºC for 12 h).

Our microscope systems can be used for collecting optical sections perpendicular to the tissue sample (z-scans) to obtain a 3D visualization of the particle distribution inside the tissue. The 3D rendering of the optical sections collected in several mouse organs is shown in S13 Fig.
